# Long-Term Complications of Multisystem Inflammatory Syndrome in Children and Adults Post-COVID-19: A Systematic Review

**DOI:** 10.3390/ijms262110695

**Published:** 2025-11-03

**Authors:** Sanish Varghese, Ibrahim Al-Hassani, Ubaida Al-Aani, Noor J. Rob, Sara Al-Mannai, Aayami Jaguri, Reel A. Yousif, Aisha Al-Mulla, Fathima F. Palayangal, Sa’ad Laws, Dana Al-Ali, Dalia Zakaria

**Affiliations:** 1 Department of Medical Education, Weill Cornell Medicine-Qatar, Doha P.O. Box 24144, Qatar; ssv4001@qatar-med.cornell.edu (S.V.); iaa4004@qatar-med.cornell.edu (I.A.-H.); aya4003@qatar-med.cornell.edu (A.A.-M.); ffp4002@qatar-med.cornell.edu (F.F.P.); 2 Health Sciences Library, Weill Cornell Medicine-Qatar, Doha P.O. Box 24144, Qatar; sal2018@qatar-med.cornell.edu; 3 Department of Pediatrics, Sidra Medicine, Doha P.O. Box 26999, Qatar; 4 Department of Premedical Education, Weill Cornell Medicine-Qatar, Doha P.O. Box 24144, Qatar

**Keywords:** COVID-19, SARS-CoV-2, post-COVID-19 sequelae, long-COVID, COVID-19 complications, multisystem inflammatory syndrome, MIS-C, MIS-A

## Abstract

The SARS-CoV-2 pandemic has posed global medical challenges due to its ability to affect multiple organ systems. Among the post-COVID-19 complications, multisystem inflammatory syndrome has emerged as a severe condition affecting both children (MIS-C) and adults (MIS-A). This review aims to compile and analyze published data to investigate clinical characteristics, laboratory findings, and outcomes of MIS post-COVID-19. A comprehensive search of various databases was conducted to identify studies reporting MIS-related complications in pediatric and adult populations post-COVID-19 infection. Screening, data extraction, and cross-checking were performed by two independent reviewers. Only 64 studies met our inclusion criteria, and compiled results revealed that cardiac complications were the predominant manifestation followed by gastrointestinal, hematologic, neurological, and mucocutaneous involvement. Laboratory findings consistently demonstrated elevated inflammatory markers including CRP, ferritin, D-dimer, and IL-6. Most patients required hospitalization, and many needed intensive care; treatment typically involved IVIG, corticosteroids, and biologic therapies. While most patients recovered, a subset experienced persistent complications. These findings highlight the importance of early recognition, multidisciplinary management, and structured follow-up for MIS. Future research is warranted to clarify the underlying mechanisms, risk factors, and long-term outcomes associated with MIS in post-COVID-19 patients.

## 1. Introduction

Coronavirus disease (COVID-19), caused by the severe acute respiratory syndrome coronavirus 2 (SARS-CoV-2), appeared in late 2019 and swiftly became a worldwide pandemic. Although first described as a respiratory disease, it is now clear that COVID-19 impairs multiple organ systems, including the nervous, circulatory, respiratory, urinary, and reproductive systems, and in severe cases can result in organ damage requiring organ transplantation [1], sometimes with long-term consequences [2].

The multisystem issues that persist or develop following an initial SARS-CoV-2 infection constitute “Post-Acute Sequelae of COVID-19” or “Long COVID” and are estimated to have affected 65 million people globally [3]. These sequelae include a wide spectrum of long-term complications involving different organ systems such as gastrointestinal disorders, hepatic dysfunction, metabolic conditions including new-onset diabetes, cardiovascular injury and neurological/psychiatric disorders [4,5,6,7,8].

Although the exact mechanism is unknown, one of the proposed mechanisms for the underlying cause of multisystem failure and acute respiratory distress syndrome in COVID-19, aside from direct viral mediated toxicity and loss of function of the angiotensin-converting enzyme 2 receptor, is a dysregulated inflammatory reaction to the virus itself caused by a state known as “cytokine storm.” It is a harmful, rapidly evolving systemic inflammatory condition characterized by elevated circulating cytokine levels and excessive immune-cell stimulation. There is a delayed abnormal release of cytokines and a breakdown of regulatory control over proinflammatory cytokines resulting in extensive inflammation and multiple organ dysfunction [9].

One important manifestation of this dysregulated extensive immune response is multisystem inflammatory syndrome (MIS). This rare but serious hyperinflammatory disorder affects many organ systems, necessitating prompt treatment and management. MIS is most often documented in children (MIS-C), although it can also occur in adults (MIS-A) with differing clinical manifestations. The Center for Disease Control and Prevention defines multisystem inflammatory syndrome in children (MIS-C) as an illness in a person < 21 years of age with fever, clinical severity requiring hospitalization, evidence of SARS-CoV-2 infection, and evidence of systemic inflammation (C-reactive protein, CRP ≥ 3.0 mg/dL). New onset manifestations are necessary in at least two clinical categories (cardiac, mucocutaneous, gastrointestinal, and hematological). MIS-A on the other hand, is defined as an illness in a person ≥ 21 years of age hospitalized for ≥24 h with fever for ≥24 h, evidence of SARS-CoV-2 infection, evidence of systemic inflammation (CRP, ferritin, interleukin-6 (IL-6), erythrocyte sedimentation rate (ESR), and procalcitonin with at least three clinical criteria (severe cardiac illness, new-onset neurologic signs, shock, gastrointestinal symptoms, and thrombocytopenia) [10].

MIS-C in children is often characterized by fever, gastrointestinal symptoms, and cardiovascular failure, which frequently mimics Kawasaki illness or toxic shock syndrome [11]. Adults with MIS-A, however, experience more severe cardiovascular consequences, such as myocarditis and arrhythmias, as well as new-onset neurologic symptoms [12]. MIS-A patients exhibit almost similar clinical characteristics to MIS-C patients, however, the degree of cardiac failure, occurrence of thrombosis, and death rate may be greater [10].

This systematic review aims to explore the clinical presentations of MIS in both children and adults recovering from COVID-19 or during active infection and analyze the current understanding of its pathogenesis, presentation, and management strategies.

## 2. Materials and Methods

The protocol (registration number INPLASY202590034) of this systematic review was developed according to the preferred reporting items for systematic reviews and metanalysis (PRISMA) [13].

### 2.1. Information Sources and Search Strategy

This study is part of a comprehensive project looking at the long-term and severe complications of COVID-19. A comprehensive search was conducted by an information professional, and sensitivity was prioritized to retrieve all relevant studies. The following databases were searched in October 2023: PubMed, Medline (Ovid, 1946–Current), Embase (Ovid, 1974–2021), Scopus, Web of Science, Science Direct, and Cochrane Library. The search was designed around keywords and controlled vocabulary that focused on “Long COVID” and variants (see File S1 for full search details). No language or date restrictions were used. All database search results were imported into EndNote (version 19) and exported to Covidence, where duplicates were removed prior to initial screening.

### 2.2. Eligibility Criteria

No restrictions were made based on country, age or gender. Duplicates were removed, and any articles that did not have primary data, such as review articles, were excluded from the study. Furthermore, studies that were not in English were excluded. Any conference abstracts were excluded, and only full articles were included. During the full-text screening, any studies that reported long-term MIS complications post-COVID-19 in children or adults were included. The inclusion criteria related to this point included any patients who developed MIS after recovering from COVID-19. If the disorder was diagnosed after at least a month after COVID-19 diagnosis or if the study reports that anti-SARS-CoV-2 Immunoglobulin G (IgG) but not Immunoglobulin M (IgM) antibodies were detected, the study was included. The studies that reported any related diagnosis during the active COVID-19 infection were included only if the symptoms lasted for more than 12 weeks after COVID-19 diagnosis, if the patients received a treatment for the disorder for at least 12 weeks after COVID-19 diagnosis, or if the patient died before 12 weeks. Any cases of MIS that were diagnosed during the active infection of COVID-19 and fully recovered within less than 12 weeks were excluded. Patients who had a history of MIS for any reason were excluded.

### 2.3. Study Selection and Data Collection

Title and abstract screening, full-text screening, and data extraction were conducted by two independent reviewers for each study using Covidence, and disagreements were resolved by consensus.

### 2.4. Data Items

Demographic and clinical data, including age, sex, comorbidities, treatments, and outcomes, were collected. Continuous variables were expressed as mean ± standard deviation or range of results. Categorical variables were expressed as percentages.

### 2.5. Risk of Bias and Quality Assessment

The quality of the included studies was assessed using different methods depending on the type of study. The Newcastle–Ottawa Quality Assessment Scale (NOS) was used to assess the cohort studies and the scale developed by Murad et al. was used to assess the case reports and case series [14]. Quality assessment was conducted by two independent reviewers.

### 2.6. Data Analysis

The MIS reported by the included studies were classified in adults and children or adolescence based on manifestations including cardiovascular, neurological, gastrointestinal or hematological.

## 3. Results

**Figure 1:**
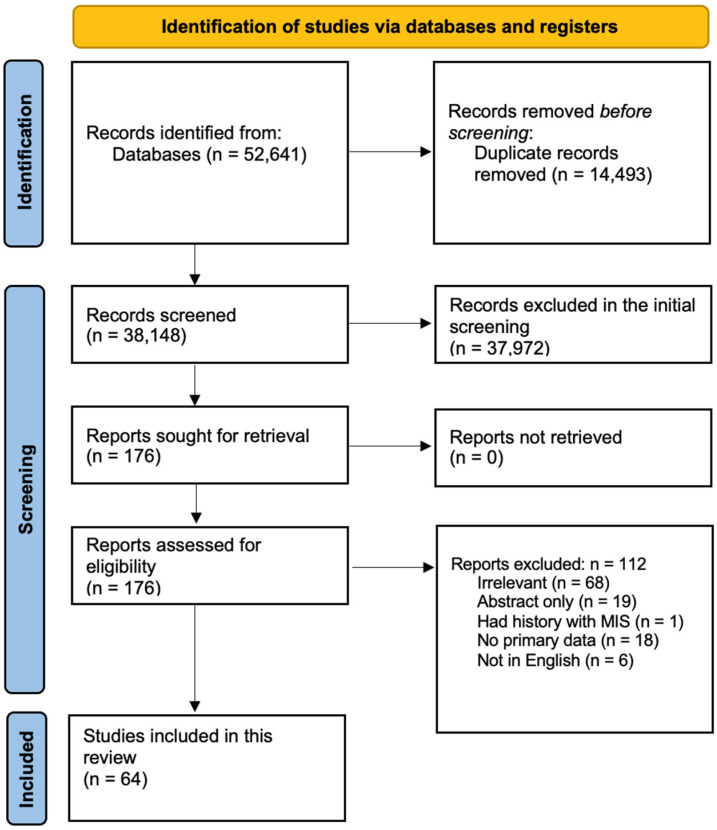
Screening and study selection protocol.

Figure 1 shows the flow diagram of the study protocol. After removing the duplicates, the titles and abstracts of 38,148 studies were screened, of which 176 were selected for full text screening. Only 64 studies met our inclusion criteria. Of the 112 excluded studies, 68 were irrelevant, 18 had no primary data, 19 were not peer reviewed or were abstracts only, 6 were not in English, and 1 had history with MIS. Supplementary Table S1 summarizes the demographic and clinical data of the included subjects as well as the quality assessment score for each study [15,16,17,18,19,20,21,22,23,24,25,26,27,28,29,30,31,32,33,34,35,36,37,38,39,40,41,42,43,44,45,46,47,48,49,50,51,52,53,54,55,56,57,58,59,60,61,62,63,64,65,66,67,68,69,70,71,72,73,74,75,76,77,78]. 

### 3.1. Types of Studies and Demographic Data

Of the 64 included studies, 39 were case reports, 7 were case series, 16 were cohort studies, 1 a cross-sectional study, and 1 a descriptive study. Among the 64 studies, 18 were from the United States of America, 11 from India, 1 from Italy, 1 from United Kingdom, 4 from Pakistan, 3 from Lebanon, 2 from Canada, 2 from France, 2 from Turkey, 2 from United Arab Emirates, and 1 each from Australia, China, Czech Republic, Georgia, Iran, Iraq, Japan, Korea, Kosovo, Kuwait, Mexico, New Zealand, Nigeria, Qatar, Romania, Slovakia, Switzerland, and Tunisia.

**Figure 2:**
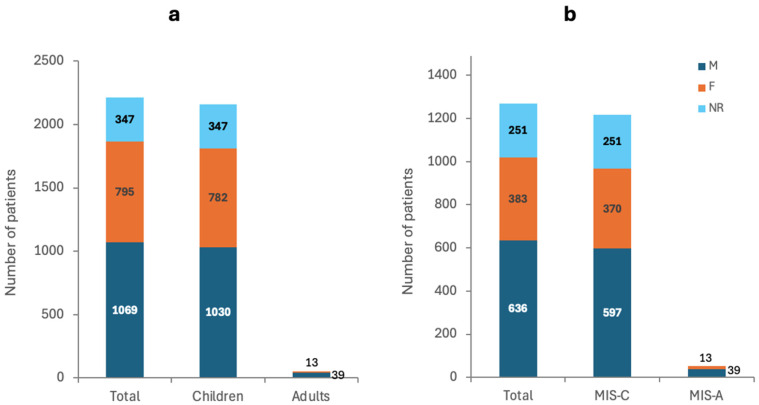
Number of patients and their gender in (**a**) COVID-19 patients and (**b**) MIS patients.

The total number of COVID-19 patients reported by the included studies was 2211, excluding Mejais et al. who reported 136,462 additional patients [65]. Mejais et al., Davies et al., and Maddux et al. did not report the gender of the reported patients [56,64,65]. However, 48.35% of the patients reported by the remaining 61 studies were males (M), 35.96% females (F), and 15.69% were not reported (NR) (Figure 2a). Furthermore, the age of the included patients ranged from 2 months to 80 years excluding Davies et al. and Maddux et al., who did not report the age of the included patients [56,64] (Supplementary Table S1).

### 3.2. Clinical Data

#### 3.2.1. Multisystem Inflammatory Syndrome in Children and Adults

**Figure 3:**
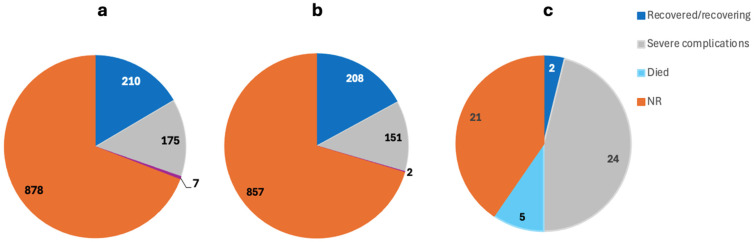
The outcome of the MIS complications in (**a**) total MIS cases, (**b**) MIS-C, and (**c**) MIS-A patients.

The 64 included studies reported a total of 3024 cases of MIS post-COVID-19. Excluding Mejais et al., Davies et al., and Maddux et al., who did not report the gender, 50.08% of the patients were M, 30.16% were F, and 19.76 were NR and all aged between 2 months and 80 years (Figure 2b) [56,64,65]. The outcome was NR for 69.13% of the patients, while 16.54% were reported as recovered/recovering, 13.78% had severe complications, and 0.55% died (Figure 3a).

#### 3.2.2. Multisystem Inflammatory Syndrome in Children

The 36 included studies reported a total of 2972 cases of MIS post-COVID-19. Excluding Mejais et al., Davies et al., and Maddux et al., who did not report the gender, 49.01% were M, 30.38% were F, and 20.61 were NR and all aged between 2 months and 17 years (Figure 2b) [56,64,65]. The outcome was NR for 70.36% of the patients, while 17.08% were reported as recovered/recovering, 12.4% had severe complications, and 0.16% died (Figure 3b).

#### 3.2.3. Multisystem Inflammatory Syndrome in Adults

The 29 included studies reported a total of 52 cases of MIS post-COVID-19. A total of 75% were M, 25% were F, and all were aged between 20 and 80 years (Figure 2b). The outcome was NR for 40.38% of the patients, while 3.85% were reported as recovered/recovering, 46.15% had severe complications, and 9.62% died (Figure 3c).

#### 3.2.4. Important Blood Markers

**Figure 4:**
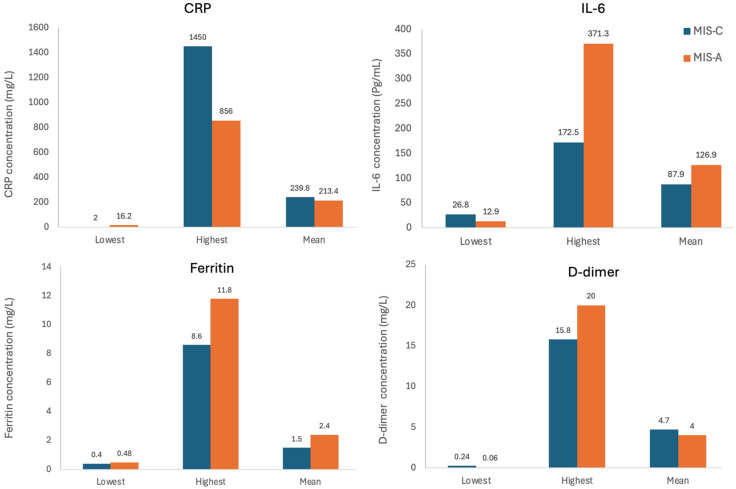
Lowest, highest, and mean levels of CRP, IL-6, ferritin, and D-dimer in the MIS-C and MIS-A groups as reported by the included studies.

The most consistently reported markers were CRP, IL-6, ferritin, and D-dimer. CRP levels were significantly elevated in both MIS-C and MIS-A groups. Mean CRP was slightly higher in children (239.76 mg/L) than adults (213.44 mg/L), both well above the reference value of <10 mg/L. IL-6 also showed marked elevation. Mean IL-6 levels in adults (126.92 pg/mL) were higher than in children (87.95 pg/mL), both exceeding the reference of <7 pg/mL. Ferritin was more elevated in adults (mean: 2421 ng/mL) compared to children (mean: 1476 ng/mL), relative to the reference range of <300 ng/mL. D-dimer showed elevated levels in both groups. Children had a slightly higher mean D-dimer (4.75 µg/mL) than adults (4.01 µg/mL), both markedly above the reference of <0.5 µg/mL (Figure 4).

#### 3.2.5. Vaccination Status

**Figure 5:**
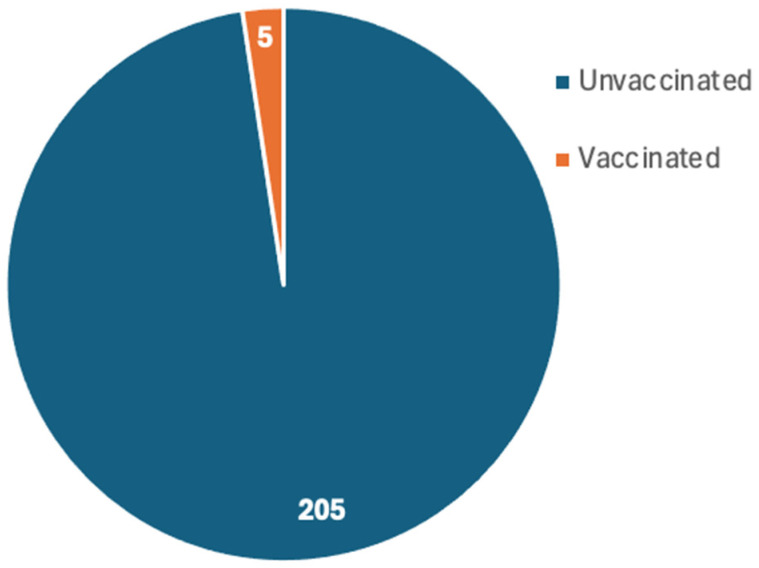
Number of vaccinated and unvaccinated patients who developed MIS post-COVID-19 infection as reported by 18 studies.

Only 18 studies reported the vaccination status [19,20,21,24,26,27,30,34,35,37,38,40,42,43,45,49,60,64], which reported a total of 210 patients who developed MIS post-COVID-19 infection. The compiled results revealed that 205 patients were unvaccinated while only 5 patients were vaccinated (Figure 5).

## 4. Discussion

This systematic review includes 64 studies from 29 different countries reporting 3024 patients who developed MIS following COVID-19 infection. MIS was observed in both children (MIS-C) and adults (MIS-A), with MIS-C accounting for the majority of cases. The most frequently affected organ systems were the cardiovascular, gastrointestinal, hematologic, and neurological systems. Given the global impact of post-COVID-19 inflammatory complications, this review aims to synthesize the available evidence on the prevalence, clinical manifestations, risk factors, and proposed mechanisms of MIS-C and MIS-A.

### 4.1. MIS-C

In total, 36 studies involving 2972 pediatric patients reported MIS-C occurring after COVID-19. The onset of MIS-C in these cases typically developed between 2 and 8 weeks after resolution of COVID-19 symptoms, with most children presenting within 3 to 4 weeks. Duration of complications varied, ranging from full resolution within 4–6 weeks to persistence of sequelae such as cardiac or neurological symptoms for 6–12 months in some cases [60].

Complications of post-COVID-19 MIS in children consist of a wide range of conditions, which can be classified into four main groups. These include cardiac, gastrointestinal, hematologic, and neurologic manifestations.

#### 4.1.1. Post-COVID-19 MIS-C Cardiac Manifestations

Cardiac involvement in children with MIS-C post-COVID-19 infection has been a significant concern, with various studies reporting numerous complications underscoring the need for vigilant monitoring of cardiac health in affected individuals.

Phirtskhalava et al. (2023)’s cohort study found the most common cardiac manifestations in MIS-C post-COVID-19 to be valve insufficiencies (mitral and tricuspid), coronary artery dilation, arrhythmias, conduction abnormalities, reduced left ventricular ejection fraction, pericardial effusion, and non-specific ST-wave changes [68,79,80]. Aziz et al. (2023) and Sai et al. (2023) reported similar manifestations. Most of the patients that developed coronary artery dilation also exhibited left anterior descending artery dilation while some showed right coronary artery dilation [51,72]. Additional investigations into establishing a link between inflammatory markers showed a significant correlation between D-dimer levels and elevated heart rate [68]. Samadi et al. (2023) reported that delayed capillary refill time was associated with a higher mortality rate and that cardiac involvement and intensive care admission were significantly higher in MIS-C patients [73]. Amongst the patients that died in the study, refractory hypotension was the main cause of mortality. Studies have shown that leukocytosis, lymphopenia, anemia, and elevated CRP and ESR levels were risk factors for increased cardiac involvement [81].

Davies et al. reported that coronary aneurysms were resolved in the majority of patients. It was found that those who continued experiencing aneurysmal changes had markedly elevated CRP levels and significantly lower median troponin levels. Persistent coronary abnormalities highlight the potential for long-term cardiac sequelae [51]. Irrespective of illness severity, when effectively treated, lab results improved swiftly throughout 6- to 10-month follow-up time [51,69]. Tamakee et al. (2023) reported MIS-C with an immune response similar to Kawasaki illness but with different characteristics like hypotensive shock and coagulopathy [76]. Al-Simaani et al. (2023)’s case presented with Kawasaki disease-like symptoms of persistent fever with tachycardia and tachypnea, non-purulent conjunctivitis, inflamed oral mucosa with swollen lips, a skin rash, a strawberry tongue, and red edematous hands and feet [49]. ECG also revealed mild dilation of the left ventricle and pericardial effusion. Many cases of MIS-C in children frequently mimic Kawasaki illness or toxic shock syndrome symptoms [54,82,83] but are distinguished by a history of COVID-19 infection [35]. Different T cell subsets and IL17-A are the difference between Kawasaki disease and MIS-C, while the similarity between them is attributed to the adverse immune reaction by auto-antibodies [84].

Morgan et al. (2023) and Appleberry et al. (2021) reported cases of cardiogenic shock and myocardial inflammation post-COVID-19 [50,67]. Despite a high initial mortality rate in the MIS-C shock subgroup, survivors generally showed clinical recovery within one month in the Aziz et al. (2023) study [51]. Cardiogenic shock was also reported by AbiNassif et al. (2021) in a child with a one-week history of generalized edema, dehydration, and signs of systemic inflammation which was secondary to left ventricular dysfunction despite treatment [44]. Pericardial effusion was reported by Ajmi et al. (2021) [47]. The child also developed renal dysfunction, and metabolic acidosis with imaging showing a pelvic ectopic kidney. Rochwerger et al. (2021) reported a case of a child who had in utero exposure to COVID-19 and developed inflammation in the coronary arteries [70]. They also presented another case who experienced chest pain and dyspnea, deteriorating to cardiac tamponade but without any effect on myocardial tissue. Draining the massive pericardial effusion in this patient led to rapid recovery.

Patients presenting with myocarditis were found to be significantly associated with severe clinical outcomes, such as needing mechanical support or being classified as New York Heart Association class IV. In addition. Out of the patients with myocarditis, 90% were also diagnosed with myocardial necrosis based on late gadolinium enhancement (LGE). Despite LGE indicating myocardial necrosis, it did not always result in persistent LV dysfunction. He et al. (2022) [78] measured myocardial injury and found the Global Longitudinal Strain in patients was decreased during the acute phase of infection. The Global Circumferential Strain was also found to be decreased in the acute phase but showed a trend towards improvement during the chronic phase [78]. It was identified that those with more severe MIS-C had decreased fractional shortening compared to milder cases.

AkaslanKara et al. (2020)’s case report presented a rare but severe complication of multiple thrombi developing after COVID-19 infection including a tricuspid valve thrombus, superior sagittal sinus thrombus, and pulmonary embolism [48]. The study highlights that high D-dimer and fibrinogen levels in MIS-C cases suggest a hypercoagulable state. Notably, even after the patient received treatment, he had significant thrombi which persisted, and the situation called for surgical management.

Most of the reported patients were treated with a combination of intravenous (IV) fluids, antipyretics, pressors, immunomodulatory drugs such as systemic glucocorticoid oral prednisolone/IV methylprednisone, intravenous immunoglobulin (IVIG), and antithrombotics such as Acetylsalicylic Acid (ASA-aspirin), vancomycin, meropenem, ceftriaxone, and esomeprazole. In severe MIS-C cases, second-line treatment using methylprednisolone is recommended for cases not responding to initial therapy with IVIG [85]. Therapeutic options for persistent MIS-C include tocilizumab, anakinra, and infliximab.

#### 4.1.2. Post COVID-19 MIS-C Gastrointestinal Manifestations

In general, a common presentation in patients with MIS-C is gastrointestinal [86]. These include but are not limited to diarrhea [45], gastroenteritis [62], and decreased liver function with elevated liver enzymes (Alanine Aminotransferase, Aspartate Aminotransferase, Gamma-Glutamyl Transferase, Alkaline Phosphatase levels) [49,67].

Campanella et al. (2022)’s [55] case was about a child with a gastrointestinal manifestation presenting with a palmar rash and petechiae on his chest. Blumberg’s sign and Rovsing’s sign were both positive [55]. Ultrasound and computed tomography (CT) revealed diffused abdominal lymphadenopathy, dense, diffuse intraperitoneal free fluid, thickening of the sigmoid colon, and signs of appendiceal inflammation. Clinicians ran a surgical exploration, which revealed diffuse turbid serous fluid, distention of the appendix, vascular congestion, and diffuse lymphadenopathy. The analysis also showed histiocytosis of sinuses and lymphoid follicles with germinal centers. Histological examination showed follicular hyperplasia and lymphoplasmacellular inflammation. It clinically resembled an acute abdomen [87]. Because the patient’s D-dimer levels were higher than usual and had a pericardial effusion, the diagnosis did not agree with just abdominal pathology. The final diagnosis included MIS-C, acute bacterial peritonitis, acute appendicitis, and acute onset lymphoproliferative disease.

Sahu et al. (2022) also presented two cases of MIS-C with gastrointestinal issues of acute appendicular and ileal perforation which ultimately had to be corrected with surgery [71]. It seems that MIS-C most commonly affects the ileum and colon. This could be due to the angiotensin converting enzyme-2 receptors found on the erythrocytes of the terminal ileum and colon leading to the inflammation of the intestine [88]. Over time, the bowel is obstructed due to the narrowing of the lumen due to reactive lymphoid hyperplasia by SARS-CoV-2. Patients with MIS-C usually present to the clinic with symptoms of appendicitis, and delay in treatment might lead to septic shock. Ajmi et al. (2021) also reported a case of a child who was hospitalized with vomiting and abdominal pain, and imaging ended up showing hepatomegaly, recto-sigmoiditis, and mesenteric lymphadenopathy [47].

#### 4.1.3. Post COVID-19 MIS-C Hematologic Manifestations

Many studies reported MIS-C patients as having low hemoglobin, platelets, white blood cells (WBC), and lymphocyte count but high neutrophils, creatinine, and blood urea nitrogen [47,49,53,72,75]. Bari et al. (2020)’s study highlighted that in severe cases the mean neutrophil–lymphocyte ratio (NLR) was higher [53]. It also found that increasing NLR was associated with poor prognosis. Bari et al. (2021)’s study also found a correlation of blood group A to severe disease [52].

Other presentations include lymphadenopathy and vasculitis. Samu et al. (2021)’s case report presented a patient with right-sided neck pain and odynophagia whose imaging of the neck revealed enlarged lymph nodes and cervical lymphadenopathy [74]. Morgan et al. (2023) also presented a child with swollen posterior cervical lymph nodes, anorexia, toothache, earache, acute kidney injury (AKI), and metabolic acidosis [67]. Fireizen et al. reported a case of AKI and respiratory symptoms that were worsening with time. The patient developed diffuse alveolar hemorrhage and severe anemia [58]. Moreover, the patient had P-Anti Neutrophil Cytoplasmic Antibody and Myeloperoxidase antibodies (indicative of vasculitis). A kidney biopsy performed on the patient showed that he had necrotizing glomerulonephritis with minimal immune complex deposition. The study confirms that the precise mechanism of autoimmune disorders is still unclear; however, the study mentions that genetic and environmental factors do play a role [89].

Thakur et al. reported a rare case of post-MIS-C Juvenile dermatomyositis (JDM). The child had contractures of both elbow and knee joints, dysphagia, weakness in the proximal muscles of the upper and lower limbs, and photosensitive rash on his extremities. Muscle biopsy showed peri fascicular atrophy and necrosis of the peri fascicular fibers, suggestive of dermatomyositis. Fine-needle aspiration of enlarged lymph nodes also showed high-grade non-Hodgkin’s lymphoma. The authors note that the severe nature of the patient’s MIS-C triggered innate immunity, leading to autoimmunity against endothelial antigens (as is characteristic of JDM). The pathogenesis likely involves B and T cells as well as the generation of cytokines like interleukin 6 and 17 [90].

#### 4.1.4. Post COVID-19 MIS-C Neurologic Manifestations

Neurologic manifestations are less commonly seen in MIS-C compared to MIS-A. Seizures were the most commonly observed neurologic manifestation in the studies analyzed, some of which were not controllable even by first-line anti-epileptics [49,61]. Appleberry et al. (2021)’s case report details Febrile Infection Related Epilepsy Syndrome (FIRES) [50]. Though mechanisms of the involvement of neurological dysregulation are not clear, the authors suspect that COVID-19 and MIS-C were non-specific contributing factors to this presentation on account of the atypical brain imaging and multi-organ pathology. Other studies have also highlighted neurological involvement in MIS-C patients below the age of 5, as is the case with this patient [91,92].

Morgan et al. (2023) reported a rare finding where a child who came in with an altered mental state, and the CT scan showed acute ischemic infarcts to the right-middle cerebral and bilateral cerebellar hemispheres with cerebral edema [67]. The patient then became anisocoric with Glasgow Coma Scale (GCS) 7. The patient was then intubated using fentanyl and vecuronium. MRI revealed severe swelling in the right-middle cerebral artery with limited swelling in the cerebellar hemispheres. The patient had to be operated on to receive an emergent right-sided decompressive craniectomy and also the placement of an extraventricular drain alongside an intracranial pressure monitor. He then received heparin continuous infusion due to elevated prothrombin time and later also began rehabilitation for his left-sided hemiparesis. He also received a cranioplasty later. He was discharged on a low-molecular-weight heparin such as enoxaprin. At follow-up, there was an improvement in the left-sided hemiparesis. The authors proposed that this patient’s arterial ischemic stroke (AIS) is likely multifactorial based on a hyperinflammatory and hypercoagulable state due to MIS-C.

Keka Sylaj et al. (2022) also reported a rare neurologic manifestation in a child with symptoms of fatigue, occasional headache, loss of appetite, and urinary tract infection with proteinuria, leukocyturia, and occasional bacteriuria [62]. The patient was found to have aseptic meningitis on cerebrospinal fluid (CSF) analysis. The authors suggest that it was a MIS-C effect in the central nervous system.

#### 4.1.5. Post COVID-19 MIS-C Other Long-Term Manifestations

Messiah et al. (2022) reported that complaints of MIS-C patients included long-term complications such as tiredness, headaches, shortness of breath, anxiety, intermittent fever, chest pain, and disrupted sleep [66]. From a behavioral lens, more than one-third of MIS-C patients reported less social connection and had longer screen time for non-educational purposes. The authors noted that the females from the patient population were nearly twice as likely to report long COVID symptoms [93], and patients from lower-resource backgrounds reported higher rates of long-term symptoms, suggesting a possible link between socioeconomic factors and symptom persistence. Mental health symptoms such as depression, anxiety, and other mood symptoms were found to be higher among children post-discharge with COVID-19 [57,94,95]. The child from Morgan et al. (2023)’s case report was also reported to no longer be sleeping well and having angry outbursts [67].

### 4.2. MIS-A

A total of 29 studies reported 52 adult cases of MIS-A. Similarly to MIS-C and MIS-A generally appeared 3–6 weeks after recovery from COVID-19, although delayed presentations as far as 12 weeks have been documented. Compared to MIS-C, complications in adults were more severe and prolonged, with cardiovascular and neurological sequelae lasting for several months in some patients.

Complications of post-COVID-19 MIS-A have a wide range of conditions including cardiovascular, neurological, hematologic, and gastrointestinal manifestations.

#### 4.2.1. Cardiovascular Manifestations

A systematic review reported that cardiovascular involvement was the most common manifestation as it occurred in 81% of the patients they enrolled in their study [96]. Cardiovascular manifestations most commonly included myocarditis and reductions in left/right ventricular ejection fraction alongside hemodynamic instability (i.e., hypotension and tachycardia).

Khokhar et al. (2022) reported an 80-year-old woman with MIS-A experiencing heart failure [34]. Similarly, Kaneko et al. (2023) [32] presented a case of a man experiencing ventricular fibrillation and who was later diagnosed with myocarditis and pericarditis, which are both commonly associated presentations with MIS-A. Kaneko et al. (2023) proposed that a potential mechanism for cardiac involvement could be that the hyperinflammatory state leads to inflammation of blood vessels, driving tissue and organ damage [32]. More specifically, Coll et al. (2021) propose two hypotheses for myocardial damage: immune system hyperactivation causing a cytokine storm consisting of macrophage and T cell infiltrates, and molecular mimicry generating cardio-specific auto-antibodies [25].

Mazumder et al. (2022) also found that a 61-year-old male with MIS-A had myocardial injury and signs of coagulopathy leading to a diagnosis of myocarditis and intravascular coagulation [37]. Similarly, a case series reported severe cardiac failure in all three patients [19]. All patients were reported to have reduced left/right ventricular ejection fraction as well as cardiogenic shock, and one patient was reported to have mild tricuspid valve insufficiency. Herrera-Garcia et al. (2022) and Zahornacky et al. (2023) both also report in their case studies that common presentations of MIS-A may also involve hypotension, tachycardia, hypoxemia, and dyspnea [31,43]. Herrera-Garcia et al. (2022) [31] also further describes that their patient also developed diastolic dysfunction and secondary endocarditis. 

Interestingly, Pettinato et al. (2022) reported a rare cardiovascular complication in a 43-year-old woman with MIS-A, whereby the patient developed spontaneous coronary artery dissection after reporting chest pain, despite the patient not having any typical risk factors for cardiovascular disease, and only initially had mild COVID-19 symptoms [40]. It was reported that the heightened inflammatory response contributed to the occurrence of the coronary artery dissection.

#### 4.2.2. Gastrointestinal Manifestations

The second most common manifestation is gastrointestinal involvement [96]. A retrospective cohort study by Das et al. reported that some of the most common gastrointestinal involvements of MIS-A included diarrhea, vomiting, and abdominal pain.

This is further corroborated by a case study on a 28-year-old male by Ghoddusi et al. (2022), which reported that the patient experienced diarrhea and vomiting with diffuse epigastric pain despite having normal bowel sounds [29]. CT also revealed mild ascending colonic thickening. Kaneko et al. (2023) also present a patient with diarrhea and vomiting but also acute enteritis [32]. Likewise, Al-Mashdali et al. (2021) found that a 21-year-old man with MIS-A experienced abdominal pain, vomiting, and diarrhea even after five days of antibiotics [16]. Alongside diarrhea and vomiting, other gastrointestinal involvements can include right hypochondriac pain and hepatomegaly [21]. The potential reason for gastrointestinal involvement may be due to the continual viral replication of COVID-19 in the gastrointestinal system [20].

#### 4.2.3. Neurologic Manifestations

Neurologic involvements were varied and ranged from mild symptoms, including confusion and changes in personality, to more severe manifestations, such as seizures and coma.

Ahsan et al. (2020) report a case of a 28-year-old man suffering from difficulty drinking fluids due to dribbling and inability to purse his lips, as well as blurring of vision and intermittent diplopia on lateral gaze [15]. He also faced difficulty processing information and had a change in behavior and personality. Although his Magnetic Resonance Imaging (MRI) was normal, he had bilateral facial nerve palsy, and an ophthalmologist reported he had bilateral optic neuritis and uveitis. The authors hypothesize that the occurrence of these neurologic symptoms even after COVID-19 infection is likely as a result of the virus’ continued presence within the nervous system, leading to inflammation and thus tissue damage.

In a more severe case, Boudhabhay et al. (2021) reported a patient who suffered a coma two days after admission with normal CSF and acute vasculitis [22]. Other neurologic involvements may also be sensory neuropathy, head-jerking, and reduced sensorium (Khokhar et al., 2022) [34]. Additionally, in Al-Mashdali et al. (2021)’s case outlined earlier, the patient also presented with unilateral Bell’s palsy [16]. It is proposed that the occurrence of Bell’s palsy is a result of molecular mimicry between viral and facial nerve antigen [97,98]. Furthermore, Nawfal et al. (2022)’s case involved a patient suffering from seizures and encephalopathy [38]. Similarly, Parpas et al. (2021) presented a case of a 67-year-old man with cognitive difficulties [39].

#### 4.2.4. Hematologic Manifestations

Hematologic complications were found in nearly all studies and generally involved elevations in various biomarkers and renal involvements.

Nearly all studies reported elevations in inflammatory markers such as CRP, ESR, troponin T, pro-B-type natriuretic peptide (pro-BNP), D-dimer, ferritin, IL-6, and procalcitonin. The elevation of these inflammatory markers and the hyperinflammatory state that follows is hypothesized to be caused by various factors including inefficient and diminished neutralizing antibody activity against SARS-CoV-2, hyperinflammation caused by spike protein’s superantigen-like motif, autoantibody mediated cell damage or inflammation, and the composition of high viral load, slow viral clearance, and delayed interferon response [99,100,101]. This may explain the development of multiple organ failure (MOF) and acute respiratory distress syndrome in MIS-A [102].

Another common reported symptom was thrombocytopenia as found in Zahornacky et al. (2023)’s case [43]. Likewise, Ahsan et al. (2020)’s patient also had thrombocytopenia as well as hypochromic, microcytic anemia, leukocytosis, and thrombocytosis [15]. Similarly, Benli et al. (2022)’s case series reported that all patients had lymphopenia [20]. Boudhabhay et al. (2021)’s 46-year-old male patient also suffered from anemia and thrombocytopenia but also faced AKI [22]. Kidney biopsy of the patient revealed renal thrombotic microangiopathy (TMA) and that the interstitial infiltrate (mainly neutrophils) was responsible for moderate acute tubular necrosis and severe tubilitis in the case. In a similar case, Parpas et al. (2021) reported that their 67-year-old patient developed leukocytosis, AKI, and severe hyponatremia [39]. Deteriorating renal function required five hemodialysis treatments which revealed acute tubular necrosis as a result of a cytokine surge. Furthermore, Lee et al. (2023) also reported a case of a 20-year-old male that suffered liver and kidney damage and had to undergo renal support [35].

Mazumder et al. (2022)’s case outlines a patient suffering from AKI with electrolyte abnormalities, elevated liver enzymes, and coagulopathy [37]. Further investigation revealed rhabdomyolysis, acute inflammatory demyelinating polyneuropathy (AIDP), and disseminated intravascular coagulation (DIC). It is believed that the cause of the rhabdomyolysis may be due to statin-induced rhabdomyolysis exacerbated by hypothyroidism or ticagrelor use [103]. This has been reported as an initial manifestation of COVID-19 or as a post-COVID-19 phenomenon, suggesting a potential link in this patient [104].

### 4.3. Possible Mechanism for Post-COVID-19 MIS-C and MIS-A

The pathogenesis of MIS following COVID-19 remains incompletely understood, but both MIS-C and MIS-A are considered post-infectious immune dysregulation syndromes rather than direct viral injuries. COVID-19 infection can trigger MIS through a complex interplay of immune dysregulation, hyperinflammation, endothelial injury, and delayed immune responses. Understanding these mechanisms is crucial for managing MIS as these complications can have long-term consequences on multiple organ systems. The proposed mechanisms for developing MIS after COVID-19 involve multiple pathways including an exaggerated post-infectious immune response, cytokine storm, molecular mimicry leading to autoimmunity, and endothelial dysfunction. Persistent immune activation even after viral clearance is thought to drive the hyperinflammatory state observed in both MIS-C and MIS-A, with differences in immune maturity and comorbidities influencing the severity and pattern of organ involvement.

#### 4.3.1. Proposed Mechanisms for Development of Post-COVID-19 MIS-C

MIS-C may not be caused by a direct viral invasion but rather may be caused by the acquired immune system as a result of SARS-CoV-2 [105]. MIS-C has a severe presentation in children, and it arises from a state of delayed hyper-inflammation induced by the virus. It is characterized by excessive inflammation, which can cause damage to organs. Severe instances of MIS-C are characterized by hypotensive shock, as well as elevated levels of inflammatory cytokines like CXCL9, IL-1, IL-6, and IL-18, increasing the risk of end-organ damage from lack of perfusion and cytotoxicity [44,106,107,108]. Sai et al. (2023)’s study reports that MIS-C usually develops 3–4 weeks following COVID-19 infection [72]. This may explain why patients often test positive for COVID-19 antibodies (indicating a past infection) but may not show signs of active infection when evaluated for MIS-C [82,109]. According to this study and similar others, children aged 6–12 years were predominantly affected, while also noting a higher prevalence in girls compared to boys [110,111,112].

Ajmi et al. (2021) discuss the possible role of SARS-CoV-2 triggering a hyperinflammatory state similar to toxic shock syndrome through possibly involving superantigens or cytokine storm, despite lacking direct evidence of SARS-CoV-2 acting as a superantigen [47,113,114]. The suggested mechanism involves an immune-mediated response triggered by SARS-CoV-2, where the virus potentially acts as a superantigen or induces an exaggerated immune response in susceptible individuals [72].

Weak antiviral T cell responses due to constrained induction of antigen-specific CD4+ and CD8+ T cells to SARS-CoV-2 in the MIS-C cohort could be a factor that may lead to unchecked viral replication in the gut. An ineffective antiviral response after SARS-CoV-2 infection may lead to reduced systemic viral clearance, hyperinflammation, and persistent antigen expression, leading to MIS-C. References [75,115] hypothesize that the hyperinflammatory state in patients with MIS-C may develop in individuals, where SARS-CoV-2 replication continues in the gastrointestinal tract after the initial infection alongside breaches in the intestinal barrier leading to the release of the superantigen-containing spike protein [100] into the blood. COVID-19 can also cause an exaggerated immune response in the host through the activation of pre-primed auto-reactive T cells, proinflammatory mediators, and molecular mimicry. The aforementioned can result in tissue damage which can cause subsequent multisystem organ dysfunction [89].

Analysis of patient samples showed no significant changes in interferon (IFN)-α nor IFN-λ, suggesting that type I and III interferons do not play a critical role in MIS-C pathogenesis, despite their role in fighting SARS-CoV-2 infection [116,117]. On the other hand, interferon gamma was significantly elevated, consistent with T cell activation [118,119]. Serum B-cell activating factor (BAFF) levels were also significantly elevated in the MIS-C cohort suggesting polyclonal B-cell activation, a mechanism seen similarly in Systemic Lupus Erythematosus (SLE) [120]. In the case of SLE, it has been shown that neutrophils can lead to an increase in BAFF, augmenting the immune process. Similarly, COVID-19 and particularly MIS-C is characterized by an increase in neutrophilia. Notably, the expression of BAFF receptor (BAFFR) was suppressed in MIS-C patient subsets, likely due to the chronically elevated levels of BAFF [121,122]. Thus, MIS-C could be characterized by a dysregulated humoral immune response with the BAFF-BAFFR axis playing a significant role [63].

The underlying mechanism of in utero COVID-19 exposure triggering MIS-C is unknown; however, one possible hypothesis suggests that while the passage of the mother’s anti-COVID-19 IgG antibodies through the placenta can provide passive immunity to the fetus, it also can trigger autoantibody production, activation and secretion of proinflammatory cytokines that result in MIS-C can be caused by the aforementioned triggering of autoantibody secretion [123,124]. This mechanism of action is similar to that of neonatal lupus. Rochwerger et al. (2021) hypothesize that the mother’s infected secretion can cause intrapartum transmission leading to fetal inflammatory response syndrome (FIRS) [70].

#### 4.3.2. Potential Mechanisms for Development of MIS-A

As of now, the pathophysiology of MIS-A is unclear, however, there is a general consensus that it arises due to delayed and irregular immune responses that occur after recovery from COVID-19 infection [96]

The main theory behind the pathophysiology that is supported by various researchers is macrophage activation and expansion through antibody-dependent enhancement [125]. In antibody-dependent enhancement, when the virus binds with pre-existing non-neutralizing antibodies, Fc Gamma receptor interaction occurs during viral uptake, leading to viral proliferation, the release of proinflammatory cytokines, and the development of immune complexes which leads to inflammation.

A systematic review also outlined that another potential cause is that coronaviruses have the ability to block type I and type III interferon responses, meaning patients that had a high viral load cannot overcome nor control virus replication, leading to excessive and delayed cytokine storms. Furthermore, the authors of the systematic review outline that complement system activation may play a role as a histological study found the presence of C3c and C5b9 deposits with increased levels of sC5b9 [126].

Another theory of pathophysiology is related to superantigens. Michailides et al. (2024) note that the increased release of proinflammatory cytokines such as IL-6, IFN- γ, Tumor Necrosis Factor-α, and IL-10 lead to T cell overproduction and B-cell deficiency [127]. Enhanced T cell activation may thus trigger major histocompatibility complex-II (MHCII) to release auto-antibodies that lead to the multi-organ damage observed in MIS-A patients. Additionally, the authors note that the elevated levels also promote hypercoagulation and a hyperinflammatory state that leads to the presentation of positive anti-S IgG antibodies, which may also support another theory that S-proteins may be acting as a superantigen that causes MIS-A symptoms.

### 4.4. Important Blood Markers in MIS-C and MIS-A

Inflammatory and coagulation markers played a central role in the diagnosis and monitoring of MIS in both children and adults. Across the included studies, CRP, IL-6, ferritin, and D-dimer were the most consistently elevated biomarkers in both MIS-C and MIS-A, reflecting a hyperinflammatory and hypercoagulable state. In general, adults showed higher chronic inflammatory markers like ferritin and IL-6 values while children had slightly higher acute-phase reactants CRP and D-dimer levels. These findings correlate with the clinical manifestations reported: severe systemic inflammation, multi-organ injury, and a predisposition to thrombosis. Multiple studies have linked the degree of elevation of these markers to the severity of cardiac and neurological complications, suggesting their utility as prognostic indicators in both MIS-C and MIS-A.

Several studies have shown that children with MIS-C often presenting with markedly elevated CRP levels have associated increased risk of cardiac involvement [73]. In patients with persistent coronary aneurysms, CRP levels remained elevated for extended periods, supporting its value as a biomarker for disease progression and recovery [56]. Basu et al. 2020 reported that ferritin levels tend to normalize only after 50 days of admission, highlighting the protracted inflammatory response in MIS [54]. High D-dimer levels frequently observed in MIS-C indicate a hypercoagulable state; however, the mechanisms linking SARS-CoV-2 infection and thrombophilia remain unclear [48]. In addition, D-dimer elevation has been correlated with increased heart rate [68], suggesting a relationship between coagulopathy and cardiovascular involvement. Severe MIS is also associated with high levels of inflammatory cytokines, including IL-6, which contribute to end-organ injury and shock due to systemic inflammation [69]. This dysregulated immune response appears to be mediated by delayed and excessive cytokine release, particularly of IL-6 leading to multiorgan dysfunction [35]. Early recognition of these blood marker patterns is essential for prompt intervention needed for full recovery.

### 4.5. Effect of Vaccination

COVID-19 vaccines have played a crucial role in reducing infection rates and mitigating severe outcomes associated with SARS-CoV-2. A systematic review assessing the efficacy and effectiveness of seven major COVID-19 vaccines found that vaccination significantly decreased infection rates, disease severity, hospitalization, and mortality across various populations [128]. Another systematic review revealed that primary vaccination even offers strong and lasting protection against SARS-CoV-2 Omicron variant-associated hospitalization, severe disease, and death, and booster doses, especially mRNA-based, were shown to enhance and prolong protection against severe outcomes [129].

Only 18 studies reported the vaccination status of the reported patients. Several studies explicitly excluded vaccinated participants or were conducted in settings where patients were unvaccinated often because vaccines were not yet available during the study period. Across the 18 included studies that reported vaccination status, the majority of MIS cases occurred in unvaccinated individuals. The 18 studies reported 210 patients who developed MIS post-COVID-19 infection of which 205 patients were unvaccinated while only 5 patients were vaccinated. The high prevalence of MIS in the unvaccinated patients indicates the protective role of vaccination, which could be attributed to the reduced severity and hospitalization as well as the post-infection complications among the vaccinated individuals [128,129]

Cutfield et al. highlighted that SARS-CoV-2 vaccination is likely to substantially reduce MIS-C risk, suggesting that post-vaccine cohorts may see a lower burden of MIS compared to earlier waves [26]. Some reports documented isolated cases in vaccinated individuals: one patient received a single dose 6 weeks prior to illness [19], another had received a third dose before onset [35], and one received a single dose a year before presentation [43]. Das et al. noted that seven patients were unvaccinated, while two vaccinated patients had prior SARS-CoV-2 exposure; one unvaccinated patient recovered rapidly with supportive therapy [27].

### 4.6. Study Limitations

This systematic review has several limitations that should be considered when interpreting the findings. Firstly, many of the included studies were case reports or small case series with only a few cohort studies. While these detailed case-based reports allowed us to extract specific insights regarding prognosis, management, and individualized outcomes, they inherently lack the robustness and generalizability of larger, more comprehensive studies. Consequently, it was not possible to draw broad conclusions regarding the incidence, prevalence, and severity of MIS complications following COVID-19 infection. Secondly, there was heterogeneity in how MIS-C and MIS-A were defined, as diagnostic criteria and thresholds for laboratory markers varied across studies. This inconsistency may have affected comparability and the ability to draw precise conclusions. Moreover, the duration and quality of follow-up were highly variable; some studies reported only in-hospital outcomes whereas others included months of post-discharge follow-up. This lack of uniformity made it difficult to accurately assess the long-term progression and resolution of MIS-related complications.

Several studies were also ambiguous in reporting whether patients were recovering, fully recovered or recovering with complications. This ambiguity occasionally required interpretation during data categorization which may have influenced some calculations or conclusions. In addition, data distinguishing complications caused by MIS from those attributable to pre-existing conditions or the acute phase of COVID-19 were limited, making it challenging to isolate the specific impact of MIS and elucidate the mechanisms by which COVID-19 leads to these outcomes. Vaccination status was inconsistently reported, likely because many studies were conducted prior to vaccine rollout or involved patients that were ineligible for vaccination at the time. This limitation hinders the ability to assess the potential protective role of vaccination against MIS. Finally, while the exclusion of conference abstracts was necessary to maintain data quality and avoid duplication, this approach may have reduced the breadth of available evidence.

In conclusion, while our systematic review provides valuable insights into MIS complications following COVID-19 from diverse geographic regions, these limitations underscore the need for more comprehensive prospective multicenter studies to better define the trajectory and outcomes of MIS in both children and adults.

## 5. Conclusions

MIS is a complex, multifactorial complication that arises after COVID-19 infection and affects both children (MIS-C) and adults (MIS-A). This review confirms that MIS typically develops 2–8 weeks following SARS-CoV-2 infection, with MIS-C being more common but MIS-A often presenting with more severe manifestations. The risk and severity of MIS appear to be influenced by multiple factors, including age, immune status, comorbidities, and the severity of the initial COVID-19 illness. Patients who experienced severe infection requiring hospitalization or intensive care unit admission were more likely to develop significant post-infectious complications, particularly cardiac and neurological. The clinical presentation of MIS spans multiple organ systems, with cardiovascular involvement (myocarditis, coronary artery changes, arrhythmias) being the most prevalent, followed by gastrointestinal, hematological, mucocutaneous, and neurological manifestations. Elevated inflammatory and coagulation markers such as CRP, ferritin, IL-6, and D-dimer were consistently associated with disease severity and provide useful tools for diagnosis and monitoring of progression. While most patients responded to timely treatment including IVIG, corticosteroids, and biologics, a subset experienced persistent complications, highlighting the importance of structured long-term follow-up. The mechanisms driving MIS are believed to involve a delayed and dysregulated immune response, cytokine storm, endothelial injury, and autoimmunity triggered by prior SARS-CoV-2 infection.

### 5.1. Recommendations for MIS-C

Presentations may vary amongst MIS-C, but the most reported symptoms were tiredness, headache, and difficulty thinking. Female patients were twice as likely to report long symptoms as opposed to males, while children from higher resource backgrounds were less likely to report these symptoms. These findings are important for practitioners to keep in mind to help identify vulnerabilities in patient sub-populations [66].

Taking into account the prevalence of cardiac manifestations in MIS-C patients, it was recommended that admitted patients should be assessed for cardiac abnormalities regardless of their clinical manifestation when MIS-C is suspected [68]. Patients may present with elevated NT-proBNP levels prior to the appearance in cardiac changes on electrocardiogram (ECG), a potential biomarker for early detection, though additional research is required to confirm this. High-dose aspirin use in patients has shown lower mortality rate likely due to the anti-inflammatory effect [73]. Additional studies have supported the use of NT-proBNP in identification of cardiac involvement but also differentiation between MIS-C and Kawaski Disease [54].

Acute Ischemic Stroke (AIS) is a rare yet noted risk that cannot be overlooked in pediatric patients with SARS-CoV-2 infection. Causes of AIS are often multifactorial but may be linked to inflammatory and hypercoagulability states in MIS-C; this can be detected through high D-dimer and low fibrinogen levels, along with ESR and CRP inflammatory markers. Low-molecular-weight heparin, mechanical thrombectomy, and decompressive craniectomy (DC) are feasible treatment modalities for AIS [67].

### 5.2. Recommendations for MIS-A

Cutfield et al. assert that because the criteria for MIS-A are broad, ranging from elevated inflammatory markers to organ dysfunction, consultation of a multidisciplinary specialist group is recommended as well as exclusion of other infectious and inflammatory diagnoses using the Center for Disease Control and Prevention (CDC) diagnostic criteria. Based on the survival rate of 93%, they also suggest aggressive treatment and intensive care unit (ICU) support using glucocorticoids and/or IVIG [26].

Lee et al. recommend that in spite of the lack of established treatment guidelines for MIS-A, use of glucocorticoid and immunoglobulin combinations are highly effective. They also suggest the use of glucocorticoid monotherapy as an alternative option for elderly patients with cardiac dysfunction and potential fluid overload issues. Lee et al. also underscore the notion that symptoms of MIS are highly variable and do not align with the CDC criteria. Therefore, they recommend that MIS should not be ruled out on the basis that only some of the clinical criteria are met as it could lead to delayed or inadequate diagnosis and treatment [35]. Similarly to Cutfield et al., ruling out other infectious and inflammatory diagnoses is essential to diagnosing and treating MIS-A. Gawas et al. recommend that MIS-A should be considered when patients present with recent COVID-19 infection alongside MODS when the septic cause is excluded through investigation. However, they note that the presence of past COVID-19 infection may not be an absolute criterion due to mild symptoms of the primary COVID-19 infection [28].

### 5.3. Suggested Alternative Treatments for MIS-C and MIS-A

Alternatives to IVIG and corticosteroids have been postulated in MIS-C where 2/6 patients in one study were successfully treated with anakinra, interleukin 1 receptor antagonist, without steroid or immunosuppressive administration. Anakinra was administered at 2 mg/kg for 3 days and was then up-titrated to 4 mg/kg on account of increased inflammatory markers [59]. Furthermore, Su et al. reported that use of IVIG and corticosteroids might not be easily feasible in the elderly [41]. Lee et al. posits that glucocorticoid monotherapy may be an alternative in such cases where cardiac dysfunction and fluid overload is a concern. Parpas et al. express similar concerns to Su et al. [39].

Kaneko et al. suggested that local inflammation (similar to that of vasculitis) may be implicated in MIS-A organ damage, so they recommend the use of granulocyte and monocyte adsorptive apheresis (GMA). They suggested that it may help correct the balance between proinflammatory and anti-inflammatory cytokines by shifting the inflammation site from the patient’s body to an extracorporeal column. Subsequent removal of leukocytes through this process recruits immature and naïve leukocytes from bone marrow, leading to an increase in harmless granulocytes and functional regulatory T cells which produce anti-inflammatory cytokines, reducing inflammation and organ damage. They justified that their use of GMA over IVIG or glucocorticoids was because the patient had increasing cTnI after IVIG and there was concern for worsening cardiac load. Their patient’s cardiac involvement was successfully resolved after GMA treatment [32].

Gillrie et al. pointed out that neutrophil and B-cell dysregulation may be key pathophysiological factors in MIS-A in particularly with regard to microvascular coagulation. They imply that MIS-A may be a complication of a delayed inflammatory phase of severe acute COVID-19 where IgA causes neutrophil activation via NETs. They suggest the use of heparin, hirudin, or APC to prevent vascular dysfunction [30].

Boudhabhay et al. noted an interesting discovery of a biopsy-proven renal thrombotic microangiopathy (TMA) finding in a 46-year-old male patient. The patient was successfully treated using eculizumab. Based on this, they suggest the use of complement blockers as an alternative therapeutic option, as TMA may be involved in the pathophysiology of MIS-A. Their justification is that kidney involvement is frequent in COVID-19 patients, with nearly 40% of cases having abnormal proteinuria [22]. Another study on a 14-year-old with acute kidney injury (AKI) was also treated with eculizumab, and their renal symptoms resolved.

Benli et al. recommend the use of recombinant IL-1 receptor antagonist to block the cytokine cascade of MIS-A at the early stage. Successful treatment requires early identification and diagnosis of MIS to be most effective [20].

### 5.4. Vaccination

Al-Simaani et al. emphasized that hospitalization rates in unvaccinated children were twice as high as in vaccinated children, underscoring the protective role of vaccines, although local uptake was low due to hesitancy and safety concerns [49]. Maddux et al. also stressed that vaccination effectively prevents severe acute COVID-19 and MIS-C, potentially reducing post-discharge sequelae [64]. Overall, the available data consistently support vaccination as an important preventive measure against MIS-related morbidity.

Given the variability in presentation and outcomes, healthcare providers should maintain a high index of suspicion for MIS in patients presenting with new systemic inflammatory features weeks after COVID-19 infection, especially in high-risk groups. Multidisciplinary management, early initiation of immunomodulatory therapy, and regular post-discharge monitoring are critical to reducing morbidity.

This systematic review highlights the urgent need for standardized diagnostic criteria, multicenter registries, and prospective studies with longer follow-up to clarify the risk factors, immune mechanisms, and long-term outcomes of MIS. Early recognition and a coordinated care approach are essential to mitigate complications and to better inform treatment strategies for this serious post-COVID-19 syndrome. To address the need for quicker and more accurate diagnosis of MIS, future research should focus on the development of specific biomarkers and imaging tools that can reliably distinguish MIS-C and MIS-A from other inflammatory or infectious syndromes. Studies investigating early indicators such as NT-proBNP, D-dimer, and cytokine profiles, as well as exploring novel diagnostics like genetic or proteomic signatures, could significantly enhance diagnostic precision. In addition, further validation of criteria that capture the broad and variable presentations of MIS is essential to prevent misdiagnosis and delays in treatment.

## Data Availability

No new data were created or analyzed in this study. Data sharing is not applicable to this article.

## Supplementary Materials

The following supporting information can be downloaded at: https://www.mdpi.com/article/10.3390/ijms262110695/s1.

## Abbreviations

The following abbreviations are used in this manuscript:

AIS	Acute Ischemic Stroke
AKI	Acute Kidney Injury
BAFF	B-cell Activating Factor
CDC	Centers for Disease Control and Prevention
COVID-19	Coronavirus Disease 2019
CRP	C-Reactive Protein
DC	Decompressive Craniectomy
ECG	Electrocardiogram
ESR	Erythrocyte Sedimentation Rate
F	Female
GMA	Monocyte Adsorptive Apheresis
ICU	Intensive Care Unit
IL	Interleukin
Ig	Immunoglobulin
IVIG	Intravenous Immunoglobulin
M	Male
MHC	Major Histocompatibility Complex
MIS	Multisystem Inflammatory Syndrome
MIS-A	Multisystem Inflammatory Syndrome in Adults
MIS-C	Multisystem Inflammatory Syndrome in Children
NOS	Newcastle–Ottawa Quality Assessment Scale
SARS-CoV-2	Severe Acute Respiratory Syndrome Coronavirus 2
SLE	Systemic Lupus Erythematosus
TMA	Thrombotic Microangiopathy
