# Supplementary material for: Long-Term Complications of Multisystem Inflammatory Syndrome in Children and Adults Post-COVID-19: A Systematic Review
Source: Int J Mol Sci. 2025 Nov 3;26(21):10695. doi: 10.3390/ijms262110695 (PMC12608246; doi:10.3390/ijms262110695)
Supplement: Supplementary file 1 [file ijms-26-10695-s001.zip › Supplementary Table S1 Final 15_09_25.pdf]

Supplemntary Table S1: Demographic and clinical data for adults and children who developed multisystem inflammatory syndrome (MIS) post-COVID-19.

Study	Study Type<br><br>Country	N (total)<br>Gender<br>(%M or F)	MIS<br>clinical features/<br>symptoms	Age<br>Mean ±SE/<br>Median<br>(IQR)<br>(years)	Comorbidities (before COVID)	COVID-19<br>Date and duration<br><br>Severity	*Diagnosis	Blood markers	Management/<br>Treatment	Any other complications	How long follow up<br><br>Outcome	vaccination status	QA
Adamu et al [45]	Case Report<br><br>Nigeria	N=1<br>100% F	Fever<br>Conjunctivitis<br>Redness of the lips<br>Hyperaemic buccal mucosa, tongue, pharynx<br>Tenderness, swelling, desquamation of palms and soles<br>Diarrhea<br>Irritability<br>Refusal to feed<br>Poor sleep<br>Nasal discharge<br>Tender submandibular lymph node enlargement	8 months	NR	May 2020<br>Asymptomatic	3 months	ESR 85 mm/h	Artesunate<br>Coratem<br>Amoxicillin<br>Ceftriaxone<br>Prednisolone<br>Aspirin<br>Ig	NR	2 weeks	Vaccination status NR	5<br>Murad et al.
Cutfield et al. [26]	Case Report<br><br>New Zealand	N=1<br>100% M	Fever<br>Painful swollen foot, toes and erythema<br>Rash<br>Diarrhea<br>Tachycardia<br>Tachypnea<br>Dactylitis<br>Conjunctival injection<br>Pulmonary oedema<br>Cardiogenic shock<br>Myocarditis	25	NR	30 days ago<br>Uncomplicated	30 days	CRP 98 mg/dL	Prednisone<br>IVIG<br>Ceftriaxone<br>Clindamycin<br>Azithromycin<br>Cilazapril<br>Candesartan<br>Omeprazole<br>Aspirin<br>Enoxaparin<br>Steroids<br>Inotropes	NR	2 months	Unvaccinated	
								Neutrophils					

Study	Study Type<br><br>Country	N (total)<br>Gender<br>(%M or F)	MIS<br>clinical features/<br>symptoms	Age<br>Mean ±SE/<br>Median<br>(IQR)<br>(years)	Comorbidities (before COVID)	COVID-19<br>Date and duration<br><br>Severity	*Diagnosis	Blood markers	Management/<br>Treatment	Any other complications	How long follow up<br><br>Outcome	vaccination status	QA
								9.5*10^9/L					
								Ferritin 957 µg/L<br>hs					
								Troponin 12 ng/L					
								Anti-SARS-CoV-2 spike antibody >250 U/mL					
								Hgb 131 g/L					
								Platelet 261*10^9/L					
								Lymphocyte 1.6*10^9/L					
								APTT 56s					
								Prothrombin ratio 1					
								Fibrinogen 7.6g/L					
								LDH 339 U/L					
								Albumin 30 g/L					
								ALT 139 U/L					
								AST 67 U/L					
								GGT 283 U/L					
								ALP 282 U/L					
								Bilirubin 20 umol/L					
								BNP 14pmol/L					
Lee et al. [35]	Case Report<br><br>Korea	N=1<br>100%M	Fever<br>Dyspnea<br>Cough<br>Sputum<br>Body pain	80	Stable angina<br>Osteoporosis	March 8, 2022<br>7 weeks<br>Symptomatic	April 26, 2022	CRP 12.3 mg/dL	Dexamethasone<br>Moxifloxacin<br>Enoxaparin<br>Methylprednisolone	NR	3 months	Received 3rd dose November 13, 2021	

Study	Study Type<br><br>Country	N (total)<br>Gender<br>(%M or F)	MIS<br>clinical features/<br>symptoms	Age<br>Mean ±SE/<br>Median<br>(IQR)<br>(years)	Comorbiditie s (before COVID)	COVID-19<br>Date and duration<br><br>Severity	*Diagn osis	Blood markers	Management/ Treatment	Any other complications	How long follow up<br><br>Outcome	vaccination status	QA
			Weakness in hip joint<br>Heart failure<br>Pleurisy<br>Polyneuropathy<br>Pleural effusion<br>Atelectasis<br>LVEF 39%										
								ESR 84 mm/h					
								PCT 3.73 ng/mL					
								Fibrinogen 765 mg/dL					
								D-dimer 2396 ng/mL					
								Ferritin 594.6 ng/mL					
								IL-6 108.8 pg/mL					
								WBC 4960/mm^3					
								89% segmented neutrophils					
								BNP 1618 pg/mL					
								ANA 1:160					
								RF 22.9 IU/mL					
Ahsan et al. [15]	Case Report<br><br>Pakistan	N=1<br>100% M	Fever<br>Chills<br>Eating very little<br>Nausea<br>Vomiting<br>Rash<br>Pain in legs<br>Weakness<br>Swelling of foot<br>Constipation<br>Difficulty urinating	28	Thalassemia minor<br>Overweight	27 May 2020<br><br>Symptomatic	6 weeks	Hgb 8.8 g/dl	Ceftriaxone<br>Odancsetron<br>Prednisolone	NR	6 weeks	Vaccination status NR	5<br>Murad et al.

Study	Study Type<br><br>Country	N (total)<br>Gender<br>(%M or F)	MIS<br>clinical features/<br>symptoms	Age<br>Mean ±SE/<br>Median<br>(IQR)<br>(years)	Comorbidities (before COVID)	COVID-19<br>Date and duration<br><br>Severity	*Diagnosis	Blood markers	Management/<br>Treatment	Any other complications	How long follow up<br><br>Outcome	vaccination status	QA
			Withdrawn<br>Restless<br>Responding with monosyllables<br>Red eyes<br>Plethoric face<br>Fatty liver<br>Mouth Dribbling<br>Blurring of vision<br>Intermittent diplopia on lateral gaze<br>Change in behavior, personality with difficulty processing information<br>Inappropriate responses<br>Facial nerve palsy<br>Optic neuritis and uveitis										
								WBC 13600/µL					
								Neutrophil 10880/µL					
								Platelet 118000/µL					
								rGT 69 U/L					
								Albumin 2.5 g/dL					
								ESR 58 mm/h					
								CRP 13.19 mg/dL					
								Ferritin 613.9 ng/mL					
Boudhabhay et al. [22]	Case Report<br><br>France	N=1<br>100% M	Renal thrombotic microangipathy<br>HTN<br>AKI	46	Arterial HTN<br>Obesity	NR	NR	COVID-19 IgG 80 UA/mL	Eculizumab<br>Heparin	NR	182 days	Vaccination status NR	6<br>Murad et al.

Study	Study Type<br><br>Country	N (total)<br>Gender<br>(%M or F)	MIS<br>clinical features/<br>symptoms	Age<br>Mean ±SE/<br>Median<br>(IQR)<br>(years)	Comorbidities (before COVID)	COVID-19<br>Date and duration<br><br>Severity	*Diagnosis	Blood markers	Management/<br>Treatment	Any other complications	How long follow up<br><br>Outcome	vaccination status	QA
			Proteinuria<br>Low natriuresis<br>Erythema<br>Myocardial dysfunction<br>LVEF 40%<br>Pericardial effusion<br>Coma<br>Acute vasculitis						Trimethoprim<br>Sulfamethoxazole<br>Dobutamine<br>RRT<br>Ventilator				
								RBC low					
								Platelet low					
								Creatinine 169µmol/L					
								CRP 312 mg/L					
								Neutrophil 18.7 g/L					
								Elevated Troponin					
								C4 69 mg/L					
								ANA 1:160					
Phirtskhalava et al.<br>[69]	Cohort<br><br>Georgia	N=103<br>50% M	Common:<br>Fever<br>Cardiovascular issues:<br>Coronary artery dilation-<br>LAD<br>RCA<br>LMCA<br>Valvular insufficiency-<br>Mitral<br>Tricuspid<br>Heart rate abnormalities<br>Conduction abnormalities<br>ST wave changes<br>Heart block<br>Less LVEF	7 (9)	NR	NR	NR	Elevated D-Dimer	Prednisolone<br>IVIG	NR	2-6 weeks<br><br>3 months	NR	5<br>NOS

Study	Study Type<br><br>Country	N (total)<br>Gender<br>(%M or F)	MIS<br>clinical features/<br>symptoms	Age<br>Mean ±SE/<br>Median<br>(IQR)<br>(years)	Comorbidities (before COVID)	COVID-19<br>Date and duration<br><br>Severity	*Diagnosis	Blood markers	Management/<br>Treatment	Any other complications	How long follow up<br><br>Outcome	vaccination status	QA
			Pericardial effusions										
								Elevated CRP Median 145 mg/dL (Range 12-393)					
								Elevated Ferritin					
								Elevated ESR Median 42 mm/h (Range 5-72)					
								Elevated Troponin					
Singh et al. [76]	Multiple Cohort Study<br><br>USA	N=60<br>55% M	P values tested against Convalscent COVID-19 group	Broken down by cohort	NR	April 2020 - Feb. 2021	NR		Broken down by cohort	NR	NR	NR	5<br>NOS
		MIS-C<br>(n=21; 48% M)	Infilitrates 56% (p=0.5)	9.4 ±3.4		NR		WBC max $13.5*10^3$cells /µL<br>SD $8.9*10^3$<br>(p=0.04)	IVIG (95%)				
			Pleural Effusions 50% (p=0.02)					Neutrophil max 11871/µL<br>SD 8048<br>(p=0.01)	Corticosteroids (95%)				
			Depressed Mycoradial Function 67% (p<0.01)					ALC min 674/µL<br>SD 396.3<br>(p<0.0001)					
			Coronary Artery Dilation 14% (p>0.99)					Platelet count min $158.1*10^3$/µL<br>SD $81.8*10^3$<br>(p=0.03)					

Study	Study Type<br><br>Country	N (total)<br>Gender<br>(%M or F)	MIS<br>clinical features/<br>symptoms	Age<br>Mean ±SE/<br>Median<br>(IQR)<br>(years)	Comorbidities (before COVID)	COVID-19<br>Date and duration<br><br>Severity	*Diagnosis	Blood markers	Management/<br>Treatment	Any other complications	How long follow up<br><br>Outcome	vaccination status	QA
			Cardiac or Respiratory Insufficiency76%<br>($p=0.01$)					Ferritin max 1426 ng/mL<br>SD 1403<br>($p<0.01$)					
								CRP max 18.9 mg/dL<br>SD 9.1<br>($p<0.0001$)					
								ESR max 47 mm/h<br>SD 22.6<br>($p=0.05$)					
								IL-2R max 5609 pg/mL<br>SD 1905					
								IL-6 max 74.5 pg/mL<br>SD 142.7					
								Sodium min 130.6 mmol/L<br>SD 5<br>($p<0.0001$)					
								Creatinine max 1.0 mg/dL<br>SD 0.7<br>($p=0.6$)					
								ALT max 84.2 U/L<br>SD 85.4<br>($p=0.5$)					
								BNP max 2246 pg/mL<br>SD 3262<br>($p=0.07$)					
								Troponin max 1.26 ng/mL<br>SD 2.56<br>($p=0.26$)					

Study	Study Type<br><br>Country	N (total)<br>Gender<br>(%M or F)	MIS<br>clinical features/<br>symptoms	Age<br>Mean ±SE/<br>Median<br>(IQR)<br>(years)	Comorbidities (before COVID)	COVID-19<br>Date and duration<br><br>Severity	*Diagnosis	Blood markers	Management/<br>Treatment	Any other complications	How long follow up<br><br>Outcome	vaccination status	QA
		Convalescent COVID-19 (n=19; 53% M)	Infiltrates 39%	12.3 ±7		27-83 days post onset<br><br>16% Outpatient mild severity		WBC max $8.1*10^3$cells/µL<br>SD $5.73*10^3$	IVIG (5%)				
			Pleural Effusions 8%			26%<br>Inpatient<br>mild severity		Neutrophils max 5724 cells/µL<br>SD 5589	Corticosteroids (37%)				
			Depressed Mycoradial Function 0%			26%<br>Inpatient<br>moderate severity		ALC min 1621 cells/µL<br>SD 1424					
			Cornoary Artery Dilation 0%			32%<br>Inpatient<br>severe		Plateletsmin $221.8*10^3$/µL<br>SD $89.4*10^3$					
			Cardiac or Respiratory Insufficiency 32%					Ferritin max 275.8 ng/mL<br>SD 254.2					
								CRP max 3.2 mg/dL<br>SD 4.9					
								ESR max 26 mm/h<br>SD 25.9					
								Sodium min 137.7 mmol/L<br>SD 3					
								Creatinine max 1.5 mg/dL<br>SD 3.2					
								ALT max 140.3 U/L<br>SD 346.1					
								BNP max 76.4 pg/mL<br>SD 151.8					
								Troponin max 0.02 ng/mL					

Study	Study Type<br><br>Country	N (total)<br>Gender<br>(%M or F)	MIS<br>clinical features/<br>symptoms	Age<br>Mean ±SE/<br>Median<br>(IQR)<br>(years)	Comorbidities (before COVID)	COVID-19<br>Date and duration<br><br>Severity	*Diagnosis	Blood markers	Management/<br>Treatment	Any other complications	How long follow up<br><br>Outcome	vaccination status	QA
								SD 0.01					
		Control<br>(n=20; 65%<br>M)		7.45 ±3.4									
Ghoddusi et al [29]	Case report<br><br>Kuwait	N=1<br>100% M	Nausea<br>Diarrhea<br>Vomiting<br>Chills<br>Hypotension	28	None	24 days prior to presentation	Within 5 days after presentation	Leukocytes<br>$21.2*10^3$cells /µL	Lactated ringer's solution<br>Ondansetn<br>Noroepinephrine drip<br>Antibiotics<br>Vasopressr<br>Intubation<br>Steroids<br>IVIG	Severe organ damage	NR	NR	Murad et al.<br>4
								Hgb/HCt16.2 g/dL /50.0%					
								Platelets<br>$155*10^3$/µL					
								Sodium 136 mmol/L					
								ALT 38 U/L					
								AST 64 U/L					
								CRP 4.2 mg/dL					
								D-dimer<br>1.122 µg/mL					
								ESR 97 mm/hr					
Al-Simaani et al. [49]	Case report<br><br>Iraq	N=1<br>100% M	Shock<br>Rash<br>Conjunctivitis<br>Vomiting<br>Diarrhea<br>GI symptoms<br>Cardiac dysfunctin<br>Seizures	10	NR	NR<br>Contact with known COVID-19 patients 4 weeks prior to admission	2nd day of admission	Hgb 9.6 g/dL – Low	Antibiotics<br>Dexamethosone<br>Phenobarbital<br>IvIg<br>Esomeprazole<br>Aspirin	NR	3 weeks - all lab values returned to normal except CRP which took 4 weeks	Nonvaccinated	Murad et al.<br>5

Study	Study Type<br><br>Country	N (total)<br>Gender<br>(%M or F)	MIS<br>clinical features/<br>symptoms	Age<br>Mean ±SE/<br>Median<br>(IQR)<br>(years)	Comorbidities (before COVID)	COVID-19<br>Date and duration<br><br>Severity	*Diagnosis	Blood markers	Management/<br>Treatment	Any other complications	How long follow up<br><br>Outcome	vaccination status	QA
								Platelets $15*10^9$/L - Low					
								WBC 2.4 × 10^9/L					
								Neutrophil 67%					
								Lymphocyte 29%					
								ALT 212 U/L					
								AST 387 U/L					
								LDH 462 U/L					
								GGT 166 IU/L					
								ALP 212 U/L					
								Albumin 3.5 g/dL					
								Bilirubin 0.84 mg/dL					
								Creatinine 1.4 mg/dL					
								BUN 50 mg/dL					
								CRP 18.3 mg/dL					
								D-dimer 5120 ng/mL					
								S. ferritin 400 ng/mL					
								Troponin T 0.44 ng/mL					
								PCT 88.3 ng/mL					
Gawas et al. [28]	Case series<br><br>India	N=4<br>75% M		All info on a case by case basis									Murad et al.<br>6

Study	Study Type<br><br>Country	N (total)<br>Gender<br>(%M or F)	MIS<br>clinical features/<br>symptoms	Age<br>Mean ±SE/<br>Median<br>(IQR)<br>(years)	Comorbidities (before COVID)	COVID-19<br>Date and duration<br><br>Severity	*Diagnosis	Blood markers	Management/<br>Treatment	Any other complications	How long follow up<br><br>Outcome	vaccination status	QA
		N=1<br>100% M	Hypotension<br>Vomiting<br>Sweating<br>Generalized weakness<br>Acute dyspnea	29	None	4 weeks prior to admission (mild)	Within 72 hours of admission	Hgb 5.8 g/dL	Antibiotics<br>LMWH<br>Seroids<br>IVIG<br>Dobutamine<br>Norepinephrine	NR	2 weeks	NR	
								TLC 16100/mm^3					
								Platelets 100000/mm^3					
								TBili 1 mg/dL					
								AST 32 IU/L					
								ALT 43 IU/L					
								BUN 27 mg/dL					
								Creatinine 0.81 mg/dL					
								Albumin 3.49 mg/dL					
								D-dimer 3480 ng/mL					
								Ferritin 2325.1 ng/mL					
								LDH 288 U/L					
								Troponin I 0.107 ng/L					
								CRP 890 mg/L					
		N=1<br>100% M	Generalized weakness<br>Hypotension<br>Nausea<br>Vomiting	49	DM for 5 years	3 weeks prior to presentation (mild)	Within 48 hours of presentation	Hgb 9.78 g/dL	Antibiotics<br>LMWH<br>Steroids<br>IVIG<br>Dobutamine<br>Norepinephrine	NR	NR	NR	

Study	Study Type<br><br>Country	N (total)<br>Gender<br>(%M or F)	MIS<br>clinical features/<br>symptoms	Age<br>Mean ±SE/<br>Median<br>(IQR)<br>(years)	Comorbidities (before COVID)	COVID-19<br>Date and duration<br><br>Severity	*Diagnosis	Blood markers	Management/<br>Treatment	Any other complications	How long follow up<br><br>Outcome	vaccination status	QA
								TLC 8240/mm^3					
								Platelets 218000/mm^3					
								TBili 0.5 mg/dL					
								AST 88 IU/L					
								ALT 110 IU/L					
								BUN 26 mg/dL					
								Creatinine 0.96 mg/dL					
								Albumin 2.1 mg/dL					
								D-dimer 7749 ng/mL					
								Ferritin 9876 ng/mL					
								LDH 482 U/L					
								Troponin I 0.056 ng/L					
								CRP 948 mg/L					
		N=1<br>100% M	Fever<br>Dyspnea Diarrhea	70	Old CVA	3 weeks prior to presentation (mild)	Within 48 hours of presentation	Hgb 8.19 g/dL	AntibioticsL<br>MWH<br>Steroids<br>IVIG<br>Dobutamine<br>Norepinephrine		3 weeks	NR	
								TLC 16500/mm^3					
								Platelets 60000/mm^3					
								TBili 1.7 mg/dL					
								AST 146 IU/L					

Study	Study Type<br><br>Country	N (total)<br>Gender<br>(%M or F)	MIS<br>clinical features/<br>symptoms	Age<br>Mean ±SE/<br>Median<br>(IQR)<br>(years)	Comorbidities (before COVID)	COVID-19<br>Date and duration<br><br>Severity	*Diagnosis	Blood markers	Management/<br>Treatment	Any other complications	How long follow up<br><br>Outcome	vaccination status	QA
								ALT 41 IU/L					
								BUN 14.12 mg/dL					
								Creatinine 0.82 mg/dL					
								Albumin 1.87 mg/dL					
								D-dimer 6351 ng/mL					
								Ferritin 1069 ng/mL					
								LDH 218 U/L					
								Troponin I 2.20 ng/L					
								CRP 826 mg/L					
		N=1<br>100% F	Bradycardia<br>Shock Sepsis<br>Blood loss<br>Preeclampsia	39	None	4 weeks prior to presentation (mild)	NR	Hgb 14.80 g/dL	Antibiotics<br>LMWH<br>Steroids<br>IVIG<br>Adrenaline<br>Norepinephrine	NR	Patient died within 48 hours	NR	
								TLC 26600 cells/mm^3					
								Platelets 330000/mm^3					
								TBili 0.84 mg/dL					
								AST 39 IU/L					
								ALT 159 IU/L					
								BUN 28 mg/dL					
								Creatinine 1.66 mg/dL					
								Albumin 2.46 mg/dL					

Study	Study Type<br><br>Country	N (total)<br>Gender<br>(%M or F)	MIS<br>clinical features/<br>symptoms	Age<br>Mean ±SE/<br>Median<br>(IQR)<br>(years)	Comorbidities (before COVID)	COVID-19<br>Date and duration<br><br>Severity	*Diagnosis	Blood markers	Management/<br>Treatment	Any other complications	How long follow up<br><br>Outcome	vaccination status	QA
								D-dimer 5493 ng/mL					
								Ferritin 5386 ng/mL					
								LDH 978 U/L					
								Troponin 10.2290 ng/L					
								CRP 760 mg/L					
Bhatt et al. [21]	Case report<br><br>India	N=1<br>100% M	Fever<br>Dry cough<br>Dyspnea<br>Cyanosis<br>Grade 3/6 murmur<br>Pleural effusion	30s	None	NR	NR	Creatinine 2.5 mg/dl	Furosemide<br>Clopidrogel<br>Digoxin<br>Sacibitril-valsartan<br>L-thyroxine	NR	6 months	Unvaccinated	Murad et al.<br>6
								BUN 74 mg/dl					
								ALT 944 U/L					
								AST 1138 U/L					
								CRP 16.2 mg/L					
								Ferritin 794.12 ng/mL					
								LDH 1674 U/L					
								D-dimer 1612 ng/mL					
								Troponin - I <10 pg/mL					
								Il-6 12.94 pg/mL					
Morgan et al. [68]	Case report<br><br>USA	N=1<br>100% M	Fever<br>Decreased liver function<br>Hypoperfusion<br>Shock<br>Metabolic acidosis	2	None	NR	1 day post admission (2 weeks post contact	WBC 17.50 K/µL	Fentanyl<br>Surgical decompression<br>Heparin<br>Tylenol	Cerbral infarction<br>Rapid neurologic deterioration	6 months	NR	5<br>Murad et al.

Study	Study Type<br><br>Country	N (total)<br>Gender<br>(%M or F)	MIS<br>clinical features/<br>symptoms	Age<br>Mean ±SE/<br>Median<br>(IQR)<br>(years)	Comorbidities (before COVID)	COVID-19<br>Date and duration<br><br>Severity	*Diagnosis	Blood markers	Management/<br>Treatment	Any other complications	How long follow up<br><br>Outcome	vaccination status	QA
							with COVID-19 positive patients)		IVIG Steroids	Post MIS-C Cardiomyopathy			
								Hgb 10.9 g/dL					
								HCt 32.20%					
								Sodium 131 mmol/L					
								CRP 26.7 mg/dL					
								ESR 53 mm/h					
								Platelets 143 K/μL					
								Creatinine 0.5 mg/dL					
								D-dimer 15823 ng/mL					
								BUN 29 mg/dL					
Klocperk et al. [64]	Multiple cohort study<br><br>Czech Republic	MIS-C:<br>N = 50<br>52% M	NR	7.8 ± 4.35	NR	NR	NR	Elevated IFN-γ levels (P= 0.0004)	Immunosuppressive therapy	NR	4 MIS-C patients had 6 month follow up	NR	7<br>NOS
								Elevated BAFF (p<0.0001)					
								ENA Ro60 Abs 11%					
								Less plasmablast (p< 0.0001)					
								100% of B cells suppressBAFFR					

Study	Study Type<br><br>Country	N (total)<br>Gender<br>(%M or F)	MIS<br>clinical features/<br>symptoms	Age<br>Mean ±SE/<br>Median<br>(IQR)<br>(years)	Comorbidities (before COVID)	COVID-19<br>Date and duration<br><br>Severity	*Diagnosis	Blood markers	Management/<br>Treatment	Any other complications	How long follow up<br><br>Outcome	vaccination status	QA
		COVID,<br>No MIS<br>N=7<br>71.4% M	NR	9.9 ± 3.9	NR	NR	NR	Elevated BAFF<br>(p = 0.0093)	None	NR	NR	NR	
		No COVID,<br>No MIS<br>N=8<br>37.5% M	NR	13.7 ± 1.9 years	NR	NR	NR	NR	None	NR	NR	NR	
Thakur et al. [78]	Case Study<br><br>India	N = 1<br>100% M	Fever<br>Joint contractures<br>Dysphagia<br>Photosensitive itching rash<br>Erythematous macules<br>Breathlessness<br>Myocarditis<br>Shock<br>Muscle weakness, tendernes<br>Malnourished<br>Pallor<br>Pitting edema<br>Enlarged cervical nodes<br>Gottron's papules<br>Dilated tortuous Capillaries, capillary dropouts.<br>Unable to stand<br>Anaemia<br>Non Hodgkin's Lymphoma<br>Perifascicular atrophy Necrosis of perifascicular fibres<br>Dermatomyositis.<br>Reduced	8 no SE or Median	No history of chronic drug intake or chronic illness.	5 months prior to second admission.<br><br>Duration: NR<br><br>Severity: NR	Diagnosed 4 months prior to second admission (1 month post COVID infection).	Hgb 10.3g/dl	Steroids (methylprednisolone)<br>Immunomodulators	NR	NR	NR	5<br>Murad et al.

Study	Study Type<br><br>Country	N (total)<br>Gender<br>(%M or F)	MIS<br>clinical features/<br>symptoms	Age<br>Mean ±SE/<br>Median<br>(IQR)<br>(years)	Comorbidities (before COVID)	COVID-19<br>Date and duration<br><br>Severity	*Diagnosis	Blood markers	Management/<br>Treatment	Any other complications	How long follow up<br><br>Outcome	vaccination status	QA
			joint spaces with calcifications in peri-articular spaces and soft tissue										
								Total Leucocyte Count (/cu mm) 12700					
								Platelet (/μL) 189 x 10^3					
								CRP (mg/l) 100					
								ESR (mm/hr) 90					
								LDH (u/L) 885					
								Uric acid (mg/dl) 5					
								CK (U/L) 1113					
Samadi et al. [74]	Cohort retrospective study<br><br>Iran	MIS-C Patients:<br>N= 73<br>72.6% M	Fever (87.7%)<br>Respiratory manifestations (19.2%)<br>GI manifestations (37.9%<br>Mucocutaneous manifestations (45.2%)<br>Neurologic manifestations (2.7%)<br>Cardiovascular manifestations (2.7%)<br>Lung infiltration patterns, atelectasis, pleural effusion (49.2%)	5.3 ± 4.1 for both MIS and non-MIS (only covid)	Underlying disease (21.8%)<br>Congenital heart disease (9.9%)<br>Surgical repair (33.3%)	Data and Duration: NR<br><br>Severity: Severe acute or critically ill	NR	%Patients with normal WBC: 58.9%	Antibiotics<br>Steroids (dexamethasone or prednisolone)<br>IVIG<br>Inotropic agents<br>Non-steroidal anti-inflammatory drugs.<br>Favipiravir<br>Diuretics.<br>Pantoprazole<br>Acetylsalicyli	NR	1 week and 3 month follow ups	NR	7<br>NOS

Study	Study Type<br><br>Country	N (total)<br>Gender<br>(%M or F)	MIS<br>clinical features/<br>symptoms	Age<br>Mean ±SE/<br>Median<br>(IQR)<br>(years)	Comorbidities (before COVID)	COVID-19<br>Date and duration<br><br>Severity	*Diagnosis	Blood markers	Management/<br>Treatment	Any other complications	How long follow up<br><br>Outcome	vaccination status	QA
			Less LVEF, decreased base excess (34.7%)						c acid<br>Intubation				
								Anemia (56.1%)					
								Normal platelet count (39.7%)					
								Normal CRP levels (8.2%)					
								Normal albumin levels (24.4%)					
		Non-MISC (COVID-19 only):<br><br>N=7<br>50% M	Fever (43.6%)<br>Respiratory manifestations (57.7%)<br>GI manifestations (35.9%)<br>Mucocutaneous manifestations (3.8%)<br>Neurologic manifestations (6.4%)<br>Cardiovascular manifestations (1.3%)<br>Hypoxemia(66.2%)<br>Less LVEF (11.1%)	Reported in cell above	Reported in cell above	Data and Duration: NR<br><br>Severity: Severe acute	NR	%patients with normal WBC 44.9%	Antibiotics<br>Steroids (dexamethasone or prednisolone)<br>IVIG<br>Inotropic agents<br>Non-steroidal anti-inflammatory drugs.<br>Favipiravir<br>DIuretics<br>Pantoprazole<br>Acetylsalicylic acid<br>Intubation	NR	1 week<br>3 months	NR	
								Anemia (37.2%)					
								Normal platelets (75.6%)					
								Normal CRP (41%)					

Study	Study Type<br><br>Country	N (total)<br>Gender (%M or F)	MIS clinical features/ symptoms	Age Mean ±SE/ Median (IQR) (years)	Comorbidities (before COVID)	COVID-19 Date and duration<br><br>Severity	*Diagnosis	Blood markers	Management/ Treatment	Any other complications	How long follow up<br><br>Outcome	vaccination status	QA
								Normal albumin (54.5%)					
Ciochetto et al. [24]	Case Report<br><br>USA	N = 2<br>50% M		24 ± 4.24									5<br>Murad et al.
			Fever<br>Chills<br>Chest pain<br>Shortness of breath Airspace opacity<br>Tachycardia<br>Conjunctivitis.<br>Erythematous oropharyn<br>Macular rash<br>TachycardiShort PR, diffuse ST, T wave abnormalities		Patient 1 (F): None	COVID-19 diagnosed a month prior to presentation. Duration of infection was 2 weeks.<br><br>COVID-19 Severity was mild	MIS detected upon admission (24 days post initial infection). Tested positive again for COVID-19 upon admission	WBC 9.4× 10^9 /L	Ceftriaxone (antibiotic)<br>Azithromycin (antibiotic)<br>650 mg aspirin<br>IVIG 2 g/kg once<br>Tocilizumab (600 mg IV once)	NR	6 weeks	Not vaccinated (vaccine not available at the time)	
								ALC: 770 cells/L					
								Platelet count: 137× 10^9 /L					
								Creatinine: 0.73 mg/dL					
								Ferritin 625.0 ng/ ml					
								CRP: 25.10 mg/L					
								D-Dimer: 1.63 mcg/mL					

Study	Study Type<br><br>Country	N (total)<br>Gender<br>(%M or F)	MIS<br>clinical features/<br>symptoms	Age<br>Mean ±SE/<br>Median<br>(IQR)<br>(years)	Comorbidities (before COVID)	COVID-19<br>Date and duration<br><br>Severity	*Diagnosis	Blood markers	Management/<br>Treatment	Any other complications	How long follow up<br><br>Outcome	vaccination status	QA
								Troponin level: 24 ng/L					
								BNP: 5300 pg/ mL					
			Fever<br>Headache<br>Vomiting<br>Abdominal pain.<br>Shortness of breath<br>Multi-organ failure<br>Lung infiltrates<br>Conjunctival injection<br>Tender abdomen<br>Tachycardia<br>ST, T wave abnormalities in the anterolateral leads<br>Low LVEF<br>Global hypokinesi		Patient 2 (M): None	COVID-19 infection date: upon admission; duration: NR<br><br>Covid-19 Severity: Severe	MIS diagnosed during infection	Serum creatinine of 2.06 mg/ dL.	IV ceftriaxone<br>Azithromycin<br>Vancomycin<br>Cefepime<br>Aspirin<br>IVIG<br>Vasopressor support (norepinephrine)<br>Steroids (prednisone - Initially 30 mg twice a day for 5 days then 10 day taper)	NR	NR	Not vaccinated (vaccine not available at the time)	
								WBC 16.5× 10^9 /L					
								Platelets 173× 10^9 /L					
								SARSCoV-2 IgG positive					
								ALT 75 U/L					
								AST 47 U/L					
								DBili 3.9 mg/dL					
								TBIli 5.9 mg/dL					
								ALP 113 mg/dL					

Study	Study Type<br><br>Country	N (total)<br>Gender<br>(%M or F)	MIS<br>clinical features/<br>symptoms	Age<br>Mean ±SE/<br>Median<br>(IQR)<br>(years)	Comorbidities (before COVID)	COVID-19<br>Date and duration<br><br>Severity	*Diagnosis	Blood markers	Management/<br>Treatment	Any other complications	How long follow up<br><br>Outcome	vaccination status	QA
								Troponin 782 ng/L					
								BNP 50,640 pg/mL					
								Ferritin 671 ng/mL.					
								D-Dimer 4.18.					
								CRP 29.20 mg/dL					
Appleberry et al. [50]	Case report<br><br>USA	N= 1<br>%M = 100%	Febrile Seizures<br>Shock<br>Restricted diffusion throughout gray and white matter<br>Decreased neuronal integrity with reduced N-acetylaspartate peaks<br>Developmental regression<br>Dysphagia<br>Diffuse hypertonia<br>Clonus	1 year (No mean or SE because N=1)	None (family history of febrile seizures on both maternal and paternal sides).	COVID Date: suspected to be 1 month prior to admission (exposure to virus at that time and presentation of flu-like symptoms);<br>Duration: NR<br>Covid-19 severity: NR	MIS diagnosed 1 month post infection:	WBC 2.26 K/µl	Intubation.<br>Fosphenytoin<br>Levetiracetam<br>Phenobarbital<br>Midazolam<br>Vasopressors<br>Empirically treated for tickborne illnesses<br>Treated for Citrobacter tracheitis<br>Pulse steroids<br>IVIG<br>Gastrostomy tube	NR	NR	NR	5<br>Murad et al.
								ALC 0.48 K/µl					
								Platelets 105 K/µl					
								CRP 5.7 mg/dl					
								PCT 59.54 ng/ml					
								Ferritin 452.4 ng/					
								PT 17.5 se					

Study	Study Type<br><br>Country	N (total)<br>Gender<br>(%M or F)	MIS<br>clinical features/<br>symptoms	Age<br>Mean ±SE/<br>Median<br>(IQR)<br>(years)	Comorbidities (before COVID)	COVID-19<br>Date and duration<br><br>Severity	*Diagnosis	Blood markers	Management/<br>Treatment	Any other complications	How long follow up<br><br>Outcome	vaccination status	QA
								PTT 47 se					
								D-dimer 2.34 µg/ml					
								Troponin 0.045 ng/l					
								BNP 1,924 pg/ml					
								LDH 711 unit/L					
								AST 208 unit/L					
								ALT 85 unit/L					
								Albumin 3.1 g/dl					
								Creatinine 0.3 mg/dl					
Davies et al. [56]	Cohort<br><br>UK	N = 68<br>Gender= NR	Aneurysms did not resolve: 6<br>Bright coronaries did not resolve: 1	NR	NR	NR	NR	Abnormal CRP (3%)	IVIG<br>Steroids<br>Biological agent	NR	>50 days	NR	6<br>NOS
								Abnormal D-dimer (3%)					
								Abnormal troponin (2%)					
								Normal lymphocytes					
								Normal neutrophils					
								Normal platelets					
								Normal creatinine					
								Normal ferritin					
								Normal ALT					
Messiah et al. [67]	Retrospective electronic health	MIS-C Patients:<br><br>N= 26	Long term complications (42.3%)<br>Headaches	MIS-C Patients:<br>9.08 ±4.86	NR	NR	NR	NR	NR	NR	<30 days<br>30-60 days<br>60-120 days	NR	8<br>NOS

Study	Study Type<br><br>Country	N (total)<br>Gender<br>(%M or F)	MIS<br>clinical features/<br>symptoms	Age<br>Mean ±SE/<br>Median<br>(IQR)<br>(years)	Comorbidities (before COVID)	COVID-19<br>Date and duration<br><br>Severity	*Diagnosis	Blood markers	Management/<br>Treatment	Any other complications	How long follow up<br><br>Outcome	vaccination status	QA
	records review<br><br>USA	50% M	(15.4%)<br>Difficulty concentrating (15.4%)<br>Shortness of breath (11.5%)<br>Anxiety (11.5%)<br>Fever (7.7%)<br>Chest pain (7.7%)<br>body aches (7.7%)<br>Hair loss (7.7%)<br>disrupted sleep (7.7%)<br>Other symptoms (7.7%)<br>Joint pain, rash, heart palpitaton, depresion, smell/taste change, rash (3.8%)								120 days		
		Non-MIS-C Patients:<br>N= 286<br>53.15% M<br><br><br>N= 26<br>50% M	Long term complications (20%)<br>Headache (15.4%)<br>Difficulty concentrating (5.2%)<br>Shortness of breath (61.54%)<br>Anxiety (20%)<br>Fever (20%)<br>Chest pain (20%)<br>Body aches (20%)<br>Hair loss (20%)<br>Disrupted sleep (20%)<br>Other symptoms (20%)	Non-MIS-C Patients:<br>6.43 ± 5.95	NR	NR	NR	NR	NR	NR	<30 days<br>30-60 days<br>60-120 days<br>120 days	NR	

Study	Study Type<br><br>Country	N (total) Gender (%M or F)	MIS clinical features/ symptoms	Age Mean ±SE/ Median (IQR) (years)	Comorbidities (before COVID)	COVID-19 Date and duration<br><br>Severity	*Diagnosis	Blood markers	Management/ Treatment	Any other complications	How long follow up<br><br>Outcome	vaccination status	QA
			Joint pain, rash, palpitaton, depresion, smell/taste change,<br>rash (20%)<br>Tiredness (4.5%)<br>Cough (5.6%)										
Reiff et al. [70]	Cohort retrospective study<br><br>USA	MIS-C: N=137<br>64% M	Depressed LVEF (26%)	Median (IQR): 9 (5-12)	Patients presented comorbidities (26%) (most common comorbidity was asthma)	Date and duration: NR	31% diagnosed with MIS during infection.	Median sodium level: 132 mmol/L	IVIG<br>Steroids<br>Vasopressor<br>Aspirin<br>Enoxaparin<br>Anakinra.<br>Infliximab	NR	1-2 weeks<br>1-2 months<br>6-8 months	NR	6<br>NOS
								Median Creatinine 0.685 mg/dL					
								Median albumin 2.5 g/dL					
								Median AST 47 U/L					
								Median ALT 41.3 U/L					
								Median WBC (10^3/uL): 18.04					
								Median ALC (10^3/uL) 0.86					
								Median Hgb 8.95 g/dL					
								Median platelet count (10^3/uL) 174 (min)					
								Median BNP (pg/mL) 798.1					

Study	Study Type<br><br>Country	N (total)<br>Gender<br>(%M or F)	MIS<br>clinical features/<br>symptoms	Age<br>Mean ±SE/<br>Median<br>(IQR)<br>(years)	Comorbidities (before COVID)	COVID-19<br>Date and duration<br><br>Severity	*Diagnosis	Blood markers	Management/<br>Treatment	Any other complications	How long follow up<br><br>Outcome	vaccination status	QA
								Median Troponin (ng/mL) 0.13					
								Median Fibrinogen (mg/dL) 591 (Max)					
								Median D-Dimer DDU (ng/mL) 1915					
								Median CRP (mg/dL) 20.25					
								ESR (mm/hr) 51 (initial)					
								Ferritin (ng/mL) 693					
			Mild MIS:<br>Fever (100%)<br>respiratory symptoms (29%)<br>cough (27%)<br>Shortness of Breath (6%)<br>GI Symptoms (83%)<br>vomiting (62%)<br>diarrhea (33%)<br>abdominal pain (48%)<br>rash (62%)<br>Conjunctivitis (67%)<br>Mucosal Changes (37%)<br>neck pain (29%)<br>headaches (31%).<br>AMS (6%)		Mild MIS:<br>Asthma (75%)<br>Congenital heart disease (6.25%)<br>HTN (6.25%)<br>Leukemia/Lymphoma in remission (6.25%)<br>Precocious Puberty (6.25%)	Severity: Mild (N=52)	In first cell (above)	In cell above	In first cell (above)	NR	1-2 weeks<br>1-2 months<br>6-8 months	NR	
			Moderate:<br>Fever (100%)<br>respiratory symptoms (14%)		Moderate MIS:<br>Asthma (69.2%)	Severity: Moderate (N=44).	In first cell	In first cell	In first cell	NR	1-2 weeks<br>1-2 months<br>6-8 months	NR	

Study	Study Type<br><br>Country	N (total)<br>Gender<br>(%M or F)	MIS<br>clinical features/<br>symptoms	Age<br>Mean ±SE/<br>Median<br>(IQR)<br>(years)	Comorbidities (before COVID)	COVID-19<br>Date and duration<br><br>Severity	*Diagnosis	Blood markers	Management/<br>Treatment	Any other complications	How long follow up<br><br>Outcome	vaccination status	QA
			cough (11%)<br>Shortness of Breath (7%)<br>GI Symptoms (93%). Vomiting (64%)<br>Diarrhea (50%)<br>abdominal pain (75%)<br>rash (52%)<br>Conjunctivitis (59%)<br>Mucosal Changes (39%)<br>neck pain (39%)<br>headaches (27%).<br>AMS (2%)		Autism (23.1%)<br>Congenital heart disease (7.7%)								
			Severe:<br>Fever (93%)<br>respiratory symptoms (15%)<br>cough (10%)<br>Shortness of Breath (10%)<br>GI Symptoms (98%)<br>Vomiting (78%)<br>diarrhea (56%)<br>abdominal pain (63%)<br>rash (44%)<br>Conjunctivitis (61%)<br>Mucosal Changes (44%)<br>neck pain (34%)<br>headaches (29%)<br>AMS (15%)		Severe MIS:<br>Asthma (57%)<br>HTN (14.2%)<br>Ex-29 wga w/chronic lung dx (14.2%)<br>Type 1 DM (14.2%)	Severity:<br>Severe (N=41).	In first cell	In first cell	In first cell	NR	1-2 weeks<br>1-2 months<br>6-8 months	NR	
Maddux et al. [65]	Multicenter prospective cohort study	MIS-C:<br><br>N= 203 (total) but reported on	Fatigue (11.3%)<br>Shortness of breath (2.5%)<br>Cough (2.5%)	NR but reported:<br>4.4% < 2 years.	Pre-existing respiratory conditions (11.9%, out of which 89%	NR	NR	NR	NR	NR	2 to 4 months	No patients were vaccinat	7<br>NOS

Study	Study Type<br><br>Country	N (total)<br>Gender (%M or F)	MIS<br>clinical features/ symptoms	Age<br>Mean ±SE/ Median (IQR) (years)	Comorbidities (before COVID)	COVID-19<br>Date and duration<br><br>Severity	*Diagnosis	Blood markers	Management/ Treatment	Any other complications	How long follow up<br><br>Outcome	vaccination status	QA
	USA	160 out 203 only<br><br>N=160<br>58.1% M	Headaches (7.5%).<br>Myalgia (3.1%)<br>Fever (0.6%)<br>Diarrhea (1.3%)<br>Sore throat (0.6%)<br>Persistent symptoms (20%)<br>No return to same level of activity (21.3%)<br>Not able to exercise as much as before (14.4%)<br>Sleeping more than usual (7.5%)<br>Unable to get work done (3.8%)<br>unable to focus (3.8%)<br>Unable to eat or drink without assistance (0.6%)	12.5% 2-5 years.<br>46.2% 5-13 years<br>36.9% 13-21 years	had isolated asthma)<br>Gastrointestinal or hepatic conditions (2%)<br>Neurological or neuromuscular conditions (4%)<br>Endocrine or metabolic conditions excluding obesity (5%)<br>Cardiovascular conditions (2%)<br>Hematologic conditions (1%)<br>Oncologic or immunosuppressive condition (<1%)<br>Renal or urologic conditions (1%)<br>Obesity (31.2%)								
		Non-MIS-C (Acute COVID-19 only)<br><br>N=155 (total) but reported on 119 out 203 only	Fatigue (14.3%)<br>Shortness of breath (9.2%)<br>Cough (9.2%)<br>Headaches (8.4%).<br>Myalgia or body aches (5%)<br>Fever (2.5%)<br>Anosmia (2.5%)	NR but reported:<br>< 2 years (24.4%)<br>2-5 years (6.7%)<br>5-13 years (20.2%)<br>13-21 years (48.7%)	Pre-existing respiratory conditions (30.3%, out of which 89% had isolated asthma)<br>Gastrointestinal or hepatic	COVID-19 Date and Duration: NR<br><br>COVID-19: Severe acute	NR	NR	NR	NR	2 to 4 months	No patients were vaccinated	

Study	Study Type<br><br>Country	N (total)<br>Gender<br>(%M or F)	MIS<br>clinical features/<br>symptoms	Age<br>Mean ±SE/<br>Median<br>(IQR)<br>(years)	Comorbidities (before COVID)	COVID-19<br>Date and duration<br><br>Severity	*Diagnosis	Blood markers	Management/<br>Treatment	Any other complications	How long follow up<br><br>Outcome	vaccination status	QA
		49.6% M	Diarrhea (1.7%)<br>Sore throat (1.7%)<br>Aguesia (0.8%)<br>Vomiting (0.8%)<br>Persistent symptoms (22.7%)<br>No return to same level of activity (14.3%)<br>not able to exercise as much as before (6.7%)<br>Sleeping more than usual (6.7%)<br>unable to get work done (4.2%)<br>Unable to focus (2.5%)<br>Unable to eat or drink without assistance (1.7%)		conditions (26%)<br>Neurological or neuromuscular conditions (24%)<br>Endocrine or metabolic conditions excluding Obesity (20%)<br>Cardiovascular conditions (9%)<br>Hematologic conditions (11%)<br>Oncologic or immunosuppressive condition (6%)<br>Renal or urologic conditions (5%)<br>Obesity (32.8%)								
Khokhar et al. [34]	Case report<br><br>USA	N=1<br>%M=100%	Headache<br>Sore throat<br>Maculopapular rash<br>Feeling of warmth<br>Abdominal pain<br>Loose, bloody bowel movement<br>Dry mucous membrane<br>Tachycardia	20 years	Anosmia and ageusia	COVID date NR<br>COVID severity NR	Exact timing NR but occurred after infection with COVID-19	WBC 8400 cells per uL	3L nasal cannula<br>15L oxygen mask<br>ventilation<br>Intubation<br>Fluid resuscitation<br>Broad-spectrum antibiotic	NR	13 months, physical function returned to baseline	Not vaccinated for COVID	5<br>Murad et al.

Study	Study Type<br><br>Country	N (total)<br>Gender (%M or F)	MIS clinical features/ symptoms	Age Mean ±SE/ Median (IQR) (years)	Comorbidities (before COVID)	COVID-19 Date and duration<br><br>Severity	*Diagnosis	Blood markers	Management/ Treatment	Any other complications	How long follow up<br><br>Outcome	vaccination status	QA
			Abdominal palpation discomfort<br>Difficulty breathing<br>Hypotension<br>LVEF 15%<br>Less RV systolic function<br>Anuric<br>Shock liver<br>Transaminitis						coverage with vancomycin and piperacillin<br>Vasopressors<br>ECMO<br>Renal replacement<br>IVIG<br>Dexamethasone				
								Hgb 14.7g/dL					
								Hematocrt 45.1%					
								Platelets 79000/uL					
								Creatinine 2.3 mg/dL					
								ALT 1906U/L					
								AST 5146U/L					
								Lactic acid 4.0 mmol/L					
								CRP 215.9 mg/L					
								ESR 17mm/h					
								Ferritin 11826ug/L					
								D-dimer: >20.00ug/mL					
								TBili 2.7mg/dL					
								TBili 2.7mg/dL					
								ALP 83U/L					
								PT 46.1s					
								ANA <80					

Study	Study Type<br><br>Country	N (total)<br>Gender<br>(%M or F)	MIS<br>clinical features/<br>symptoms	Age<br>Mean ±SE/<br>Median<br>(IQR)<br>(years)	Comorbidities (before COVID)	COVID-19<br>Date and duration<br><br>Severity	*Diagnosis	Blood markers	Management/<br>Treatment	Any other complications	How long follow up<br><br>Outcome	vaccination status	QA
								C3-complement 150mg/dL					
								C4-complement 33mg/dL					
								Anti-MPO Ab <5.0CU					
								Anti-proteinase 3 Ab: <5.0CU					
								Anti-MPO Ab <5.0CU					
								Anti-proteinase 3 Ab <5.0CU					
Khatoon et al . [33]	Case report<br>Lebanon<br>(Patient 1)	N=1<br>100% M	Fever<br>Sore throat<br>Diarrhea<br>Myalgia<br>Arthralgia<br>Rash<br>Conjunctivitis<br>Tachychardia<br>Soft abdomen<br>AKI<br>Dyspnea<br>Hypoxemia<br>Pulmonary edema<br>Ventricular fibrillation	28 yrs	NR	NR	NR	WBC (day 0) 7300	IV hydration<br>Lasix<br>Bilevel positive airway (Bipap)<br>Intubation<br>Cardio-respiratory resuscitation	NR	Passed away	NR	4<br>Murad et al.
								WBC (day 1) 4100					
								WBC (day 2) 19000					
								Lymphocytes (day 0) 500					
								Lymphocytes (day 1) 500					
								Lymphocytes (day 2) 1700					

Study	Study Type<br><br>Country	N (total)<br>Gender<br>(%M or F)	MIS<br>clinical features/<br>symptoms	Age<br>Mean ±SE/<br>Median<br>(IQR)<br>(years)	Comorbidities (before COVID)	COVID-19<br>Date and duration<br><br>Severity	*Diagnosis	Blood markers	Management/<br>Treatment	Any other complications	How long follow up<br><br>Outcome	vaccination status	QA
								Platelets (day 0) 62600					
								Platelets (day 1) 61400					
								Platelets (day 2) 96000					
								BUN (day 0) 45					
								BUN (day 1) 60					
								BUN (day 2) 73					
								Creatinine (day 0) 4.57					
								Creatinine (day 1) 5.8					
								Creatinine (day 2) 6.7					
								ALT (day 0) 358					
								ALT (day 1) 516					
								ALT (day 2) 702					
								AST (day 1) 1358					
								AST (day 2) 3342					
								GGT (day 0) 519					
								GGT (day 1) 476					
								GGT (day 2) 500					
								ALP (day 1) 173					
								ALP (day 2) 176					
								TBili (day 0) 3.4					
								TBili (day 2) 5.2					

Study	Study Type<br><br>Country	N (total)<br>Gender<br>(%M or F)	MIS<br>clinical features/<br>symptoms	Age<br>Mean ±SE/<br>Median<br>(IQR)<br>(years)	Comorbidities (before COVID)	COVID-19<br>Date and duration<br><br>Severity	*Diagnosis	Blood markers	Management/<br>Treatment	Any other complications	How long follow up<br><br>Outcome	vaccination status	QA
								DBili (day 0) 2.5					
								Dbili (day 1) NR					
								DBili (day 2) 3.6					
								Troponin (day 2) 1.9 ng/dl					
								CRP (day 0) 32 mg/dl					
								CK (day 1) 1143					
								INR (day 1): 1.8					
	Case report<br>Lebanon<br>(Patient 2)	N=1<br>%M=100%	Fever<br>Dyspnea<br>Diarrhea<br>Abdominal pain<br>Rash<br>Chest pain	40 yrs	NR	Mild COVID-19 infection 2 months prior to admission	NR	WBC (day 0) 3700	IV steroids<br>LMWH (Lovenox)<br>Oral anticoagulation	NR	NR	NR	
								WBC (day 1) 2800					
								WBC (day 2) 5900					
								WBC (day 3) 10000					
								WBC (day 4) 10900					
								WBC (day 5) 8500					
								WBC (day 8): 17200					
								WBC (day 9) 13600					
								Lymphocytes (day 0) 300					
								Lymphocytes (day 1) 400					
								Lymphocytes (day 2) 2200					

Study	Study Type<br><br>Country	N (total)<br>Gender (%M or F)	MIS clinical features/ symptoms	Age Mean ±SE/ Median (IQR) (years)	Comorbidities (before COVID)	COVID-19 Date and duration<br><br>Severity	*Diagnosis	Blood markers	Management/ Treatment	Any other complications	How long follow up<br><br>Outcome	vaccination status	QA
								Lymphocytes (day 3) 6900					
								Lymphocytes (day 4) 5000					
								Lymphocytes (day 5) 2500					
								Lymphocytes (day 8) 3200					
								Lymphocytes (day 9) 2600					
								Platelets (day 0) 196000					
								Platelets (day 1) 133000					
								Platelets (day 2) 180000					
								Platelets (day 3) 164000					
								Platelets (day 4) 236000					
								Platelets (day 5) 238000					
								Platelets (day 8) 529000					
								Platelets (day 9) 560000					
								Creatinine (day 0) 1.19					
								Creatinine (day 1) 1.73					
								Creatinine (day 2) 1.01					
								Creatinine (day 3) 0.8					
								Creatinine (day 4) 0.78					
								Creatinine (day 5) 0.86					
								Creatinine (day 8) 0.75					
								ALT (day 0) 301					

Study	Study Type<br><br>Country	N (total)<br>Gender<br>(%M or F)	MIS<br>clinical features/<br>symptoms	Age<br>Mean ±SE/<br>Median<br>(IQR)<br>(years)	Comorbiditie s (before COVID)	COVID-19<br>Date and duration<br><br>Severity	*Diagn osis	Blood markers	Management/ Treatment	Any other complications	How long follow up<br><br>Outcome	vaccination status	QA
								ALT(day 1)<br>312					
								ALT (day 2)<br>197					
								ALT (day 3)<br>172					
								ALT (day 4)<br>186					
								ALT (day 5)<br>152					
								ALT (day 8)<br>76					
								BUN (day 0)<br>10					
								BUN (day 1)<br>16					
								BUN (day 4)<br>11					
								AST (day 0)<br>357					
								AST (day 1)<br>400					
								AST (day 2)<br>113					
								AST (day 3)<br>79					
								AST (day 4)<br>94					
								AST (day 5)<br>50					
								AST (day 8)<br>41					
								GGT (day 0)<br>342					
								GGT (day 1)<br>302					
								GGT (day 3)<br>259					
								GGT (day 4)<br>288					
								GGT (day 5)<br>244					

Study	Study Type<br><br>Country	N (total)<br>Gender<br>(%M or F)	MIS<br>clinical features/<br>symptoms	Age<br>Mean ±SE/<br>Median<br>(IQR)<br>(years)	Comorbidities (before COVID)	COVID-19<br>Date and duration<br><br>Severity	*Diagnosis	Blood markers	Management/<br>Treatment	Any other complications	How long follow up<br><br>Outcome	vaccination status	QA
								ALP (day 0) 163					
								ALP (day 1) 116					
								ALP (day 3) 166					
								ALP (day 4) 134					
								ALP (day 5) 104					
								Ferritin (day 3) 3419					
								Ferritin (day 6) 729					
								D-dimer (day 3) 6038 ng/dl					
								D-dimer (day 6) 1460 ng/dl					
								CRP (day 0) 1.7 mg/dl					
								CRP (day 1) 1.7 mg/dl					
								CRP (day 2) 4.9 mg/dl					
								CRP (day 4) 3.4 mg/dl					
								CRP (day 5) 1.3 mg/dl					
								CRP (day 9) 0.4 mg/dl					
								Troponin (day 3) 0.014 ng/dl					
								Troponin (day 4) 0.007 ng/dl					
Kashyap et al. [62]	Case report<br>India	N=1<br>100% M	Fever<br>Maculopapular rash<br>Abnormal movement	7 months old		+ve COVID-19 infection in family members 20d before admission, severity NR	Detected after infection	CRP 8.73 mg/dl	Antiepileptic drugs<br>Intubation<br>Midazolam<br>Methylpredni	NR	NR	NR	4<br>Murad et al.

Study	Study Type<br><br>Country	N (total)<br>Gender<br>(%M or F)	MIS<br>clinical features/<br>symptoms	Age<br>Mean ±SE/<br>Median<br>(IQR)<br>(years)	Comorbidities (before COVID)	COVID-19<br>Date and duration<br><br>Severity	*Diagnosis	Blood markers	Management/<br>Treatment	Any other complications	How long follow up<br><br>Outcome	vaccination status	QA
			Conjunctival congestion<br>Seizure				(duration NR)		solone therapy<br>IVIG				
								BNP 2536					
								D-dimer 1839					
								Ferritin 86.26					
Bari et al. [52]	Descriptive cross-sectional<br><br>Pakistan	N=83<br>61% M	Fever: 85%<br>Cough: 48%<br>Respiratory difficulty: 34%<br>Rhinorrhea: 12%<br>Poor feeding: 27%<br>Body aches: 14%<br>Vomiting: 22%<br>Loose motions: 19%<br>Abdominal pain: 18%<br>Seizures: 12%	7.0+/-4.3<br>(95%CI: 6.07-8.75)<br>(SD)	24% had an underlying comorbidity (not specified) and 8% were surgical cases	12% asymptomatic<br>64% mild-moderate<br>24% severe/critical	NR			6% of participants died, 4 of which were due to a comorbid condition and only one died due to MIS-C (delay in diagnosis)			6<br>NOS
		COVID-19:<br>N=60<br>Gender: NR		NR	NR	NR		Mean leucocytes 14.0 +/-12.5 *10^9/L p=0.888					
								Neutrophils 52.0 +/-22.1%					
								ALC 5.02+/-4.81 *10^9/L p<0.001					
								Mean lymphocyte 39.1 +/-21.4% p<0.001					
								Mean platelets 297.61 +/-147.3					

Study	Study Type<br><br>Country	N (total)<br>Gender<br>(%M or F)	MIS<br>clinical features/<br>symptoms	Age<br>Mean ±SE/<br>Median<br>(IQR)<br>(years)	Comorbidities (before COVID)	COVID-19<br>Date and duration<br><br>Severity	*Diagnosis	Blood markers	Management/<br>Treatment	Any other complications	How long follow up<br><br>Outcome	vaccination status	QA
								*10^9/L<br>p=0.159					
								Mean Hgb<br>10.6 +/- 2.2<br>g/dl p=0.698					
		MIS-C:<br>N=23<br>Gender:<br>NR		NR	NR	NR		Mean<br>leucocytes<br>13.6 +/-6.9<br>*10^9/L					
								Neutrophil<br>76.5 +/- 15%					
								ALC: 2.13+/-<br>0.95 *10^9/L					
								Mean<br>lymphocytes<br>18.8+/-12.8%					
								Mean<br>platelets<br>238.5 +/-<br>206.0<br>*10^9/L					
								Mean Hgb<br>10.4 +/ 1.6<br>g/dl					
Fouriki et al. [59]	Case series<br><br>Switzerland	N=6<br>83% M		9.83 +/-<br>1.25	None	NR							6<br>Murad et al.
		Patient 1<br>M	Fever<br>Diarrhea<br>Conjunctival injection<br>Lymphadenopathy<br>Extremity edema, erythema<br>Headache<br>irritability<br>Phono photophobia<br>Petechiae	5 years	None	NR	NR	CRP<br>>500mg/L	O2 support<br>Vasoactive<br>diuretics<br>Flucloxacillin for 14d<br>1 doses of 2g/kg IVIG started on day 3<br>Acetylsalicylic acid	NR	NR	NR	

Study	Study Type<br><br>Country	N (total)<br>Gender<br>(%M or F)	MIS<br>clinical features/<br>symptoms	Age<br>Mean ±SE/<br>Median<br>(IQR)<br>(years)	Comorbidities (before COVID)	COVID-19<br>Date and duration<br><br>Severity	*Diagnosis	Blood markers	Management/<br>Treatment	Any other complications	How long follow up<br><br>Outcome	vaccination status	QA
			Respiratory insufficiency<br>Pleural effusion<br>Coronary dilation<br>IVA aneurysm						treatment				
								PCT 1.67 mg/l					
								Ferritin 176 mcg/L					
								Neutrophils 8.3 G/L					
								BNP 11383 ng/L					
								IL-1Ra 2215 pg/ml					
								IL-6: <11pg/ml					
		Patient 2<br>M	Fever<br>Abdominal pain<br>Diarrhea<br>Emesis<br>Rash<br>Extermity erythema<br>Respiratory insufficiency<br>Shock<br>Encephalopathy<br>Coronary dilation<br>Cardiac dysfunction<br>LVEF 45<br>Bronchopneumonia<br>Mesenteric adentitis<br>Ascites	8 years	None	NR	NR	CRP 379 mg/L	Mechanical ventilation<br>Milirinone<br>Noradrenaline<br>Dopamine<br>Ceftriaxone+ metronidazole for 10 days<br>2 doses of IVIG (2g/kg) on days 2 and 3<br>Acetylsalicylic acid 2mg/kg<br>Anakinra for 3 days,	NR	NR	NR	

Study	Study Type<br><br>Country	N (total)<br>Gender (%M or F)	MIS clinical features/ symptoms	Age Mean ±SE/ Median (IQR) (years)	Comorbidities (before COVID)	COVID-19 Date and duration<br><br>Severity	*Diagnosis	Blood markers	Management/ Treatment	Any other complications	How long follow up<br><br>Outcome	vaccination status	QA
			Paralytic sub-ileus						4mg/kg>7 days started on day 6				
								PCT 6.4 mcg/l					
								Ferritin 912 mcg/L					
								Neutrophil count 28.42 G/L					
								N-terminal proBNP 42225 ng/L					
								IL-1Ra 14273 pg/ml					
								IL-6 <11 pg/ml					
		Patient 3<br>M	Fever>4d>40C<br>Abdominal pain<br>Diarrhea<br>Emesis<br>Respiratory insufficiency<br>Shock<br>Hepatic cytolysis<br>LVEF 45%<br>Moderate pleural effusion<br>Abdominal lymphadenopathy and ileitis	10 years	None	NR	NR	CRP 176mg/L	Mechanical ventilation<br>Milirinone<br>Dopamine<br>Noradrenaline<br>Dobutamine<br>Vasoacive diuretics<br>2mg/kg >7 days<br>Anakinra on day 3	NR	NR	NR	
								PCT 30.2 mcg/l					
								Ferritin 799 mcg/L					

Study	Study Type<br><br>Country	N (total)<br>Gender<br>(%M or F)	MIS<br>clinical features/<br>symptoms	Age<br>Mean ±SE/<br>Median<br>(IQR)<br>(years)	Comorbidities (before COVID)	COVID-19<br>Date and duration<br><br>Severity	*Diagnosis	Blood markers	Management/<br>Treatment	Any other complications	How long follow up<br><br>Outcome	vaccination status	QA
								Neutrophils 15.94G/L					
								proBNP 10894 ng/L					
								IL-1Ra 16680 pg/ml					
								IL-6 <11 pg/ml					
		Patient 4<br>M	Fever<br>Abdominal pain<br>Diarrhea<br>Emesis<br>Rash<br>Conjunctival injection<br>Extremity erythema<br>Headache<br>Irritability<br>Phono photophobia<br>Petechiae<br>Respiratory insufficiency<br>Shock<br>Meningeal enhancement sulcus centralis, postcentral region (R)	14	None	NR	NR	CRP 415 mg/L	Ceftriaxone for 4 days<br>Mechanical ventilation<br>Adrenaline<br>Noradrenaline<br>1 dose of IVIG (2g/kg) started on day 2<br>Acetylsalicylic acid<br>Methylprednisolone (2mg/kg/d) 2mg/kg<br>Anakinra for 10 days	NR	NR	NR	
								PCT 4.16 mcg/l					
								Ferritin 1316 mcg/L					

Study	Study Type<br><br>Country	N (total)<br>Gender<br>(%M or F)	MIS<br>clinical features/<br>symptoms	Age<br>Mean ±SE/<br>Median<br>(IQR)<br>(years)	Comorbidities (before COVID)	COVID-19<br>Date and duration<br><br>Severity	*Diagnosis	Blood markers	Management/<br>Treatment	Any other complications	How long follow up<br><br>Outcome	vaccination status	QA
								Neutrophil count 17.69 G/L (29.75 under steroids)					
								N-terminal proBNP 7206 (ng/L)					
								IL-6 396pg/ml					
		Patient 5<br>F	Fever<br>Abdominal pain<br>Conjunctival injection<br>Extremity edema, erythema<br>Cheilitis<br>Respiratory insufficiency<br>Shock<br>Pleural, pericardial effusion<br>Abdominal lymphadenopathy<br>AKI<br>Hepatic cytolysis	11	None	NR	NR	CRP 200 mg/L	O2 support<br>Noreadrenaline<br>Ceftriaxone<br>Clindamycin<br>Meropenem and vancomycin<br>Acetylsalicylic acid<br>Methylprednisolone<br>Anakinra<br>Tocilzumab	NR	NR	NR	
								PCT 10 mcg/l					
								Ferritin 270 mcg/L					
								Neutrophils 10 G/L					
								proBNP 2808 ng/L					
								IL-1Ra >5000 pg/ml					

Study	Study Type<br><br>Country	N (total)<br>Gender (%M or F)	MIS clinical features/ symptoms	Age Mean ±SE/ Median (IQR) (years)	Comorbidities (before COVID)	COVID-19 Date and duration<br><br>Severity	*Diagnosis	Blood markers	Management/ Treatment	Any other complications	How long follow up<br><br>Outcome	vaccination status	QA
								IL-6 90 pg/ml					
		Patient 6<br>M	Fever<br>Diarrhea<br>Emesis<br>Rash<br>Conjunctival injection<br>Headache<br>Iritability<br>Respiratory insufficiency<br>Shock<br>Encephalopathy<br>LVEF 45%<br>Systolc, diastolic dysfunction of left ventricle<br>Pleural effusion<br>Thickening of inter-lobular septas<br>Polyneuroapthy<br>Encephalopathy	11	None	NR	NR	CRP 200 mg/L	Mechanical ventilation<br>Milirinone<br>Dopamine<br>Adrenaline<br>Noreadernaline<br>Vasopressors<br>Vasoactive diuretics<br>Vasoactive corticosteroids<br>CeftriaxonIVIG<br>Acetylsalicylic acid<br>Methylprednisolone<br>Anakinra<br>Tocilzumab<br>Hydroxychloroquine	NR	NR	NR	
								PCT 50 mcg/l					
								Ferritin 1155 mcg/L					
								Neutrophil count 23 G/L					
								N-terminal proBNP >70000 ng/L					
								IL-1 antagonist					

Study	Study Type<br><br>Country	N (total)<br>Gender<br>(%M or F)	MIS<br>clinical features/<br>symptoms	Age<br>Mean ±SE/<br>Median<br>(IQR)<br>(years)	Comorbidities (before COVID)	COVID-19<br>Date and duration<br><br>Severity	*Diagnosis	Blood markers	Management/<br>Treatment	Any other complications	How long follow up<br><br>Outcome	vaccination status	QA
								receptor >5000 pg/ml					
								IL-6 516 pg/ml					
Rochwerger et al. [71]	Case report<br><br>Canada	N=2<br>50% M											5<br>Murad et al.
		N=1<br>100% M	Fever<br>Cough<br>Rhinorrhea<br>Fissured lips<br>Conjunctivitis<br>Rash<br>Right coronary artery 3-4mm (Z score of 4.7)<br>Proximal and distal left coronary artery 3 mm (Z score 5-6)	4 months old	None	Mother was ill with COVID-19 while pregnant a few weeks before delivery	After infection	Hgb 9.6 g/dL	IVIG<br>Corticosteroids<br>Aspirin	NR	NR	NR	
								Leukocytes 23.5*10^3/uL					
								Neutrophils 13.6*10^3/uL					
								Thrombocytes 534*10^3/uL					
								CRP 7.95mg/dL					
		N=1<br>100% F	Chest pain<br>Dyspnea<br>Desaturation<br>Tachycardia<br>Fever<br>Hepatomegaly<br>Pleural effusion<br>Tamponade	16 years old	None		During infection	Hgb 11.3 g/dL (admission)	Pericardial fluid drainage<br>High dose steroids<br>Colchicine	NR	NR	NR	
								WBC 13.27*10^3/uL (admission)					

Study	Study Type<br><br>Country	N (total)<br>Gender<br>(%M or F)	MIS<br>clinical features/<br>symptoms	Age<br>Mean ±SE/<br>Median<br>(IQR)<br>(years)	Comorbidities (before COVID)	COVID-19<br>Date and duration<br><br>Severity	*Diagnosis	Blood markers	Management/<br>Treatment	Any other complications	How long follow up<br><br>Outcome	vaccination status	QA
								Neutrophil 10.95*10^3/uL (admission)					
								Thrombocytes 605*10^3 thrombocytres/uL (admission)					
								CRP 17.2 mg/dL (admission)					
								Troponin <13ng/dL (day 1)					
								Fibrinogen 838mg/dL (day 1)					
								D-dimer 8475 ng/mL (1st day)					
Kaneko et al. [32]	Case report<br><br>Japan	N=1<br>100% M	Fever<br>Vomiting<br>Diarrhea<br>Edematous thickening of small intestinal wall<br>Mild splenomegaly<br>Enteritis<br>AKI<br>Rash<br>Neck pain<br>Tachypnea<br>Neck swelling<br>Conjunctival hyperemia<br>Oliguria<br>Hypotension<br>Ventricural fibrillation<br>Myocarditis	44 years	NR	NR	After infection, in hospital (exact timing not mentioned)	WBC 215*10^2 cells/uL (maximal)	Loxoprofen<br>Levofloxacin<br>Inotrope<br>HD<br>Meropenem<br>Mechanical ventilation<br>Methylprednisolone<br>IVIG<br>IV heparin<br>Oral aspirin<br>Vancomycin and tazobactam<br>Granulomonocytapheresis<br>Glucocorticoids	NR	NR	NR	7<br>Murad et al.

Study	Study Type<br><br>Country	N (total)<br>Gender<br>(%M or F)	MIS<br>clinical features/<br>symptoms	Age<br>Mean ±SE/<br>Median<br>(IQR)<br>(years)	Comorbidities (before COVID)	COVID-19<br>Date and duration<br><br>Severity	*Diagnosis	Blood markers	Management/<br>Treatment	Any other complications	How long follow up<br><br>Outcome	vaccination status	QA
			Pericarditis										
								Platelets 13.2 *10^4/uL (max)					
								Creatinine: 9.46mg/dL (max)					
								CRP 33.41 mg/dL (max)					
								Troponin I 7124 pg/mL (max)					
Fireizen et al. [58]	Case report - USA	N=1<br>%M=100	NR: MIS	17 yrs old	Obesity<br>Asthma	NR	Post recovery period p-ANCA diagnosed	Hgb 5.5g/dL	Methylprednisolone<br>Plasmapheresis for 5 cdays<br>Cyclophosphamide	Cough<br>Fatigue<br>Dyspnea<br>Amber-colored urine<br>Diff Alveolar hemorrhage<br>AKI	NR	NR	6<br>Murad et al.
								Hematocrit 16.8%					
								BUN/Cr 30/1.52 mg/dl					
Samu et al. [75]	Case report<br><br>India	N=1<br>%M=100	Fever<br>Right-sided neck pain<br>Odynophagia	17yrs	NR	NR	upon admission after labs	Platelets 7800 cells/cumm	Paracetamol<br>IVIG<br>AntibioticsIV<br>Steroids	NR	NR	NR	6<br>Murad et al.
								CRP 241 mg/L					
								D-dimer 6501.71 ng/ml					
								FerritiN 2474 ng/ml					
Doshi et al. [57]	Cohort<br><br>USA	N=7 million commercially insured children<br>%M=NR		0–17 years of age	NR	NR	NR	NR	NR	Cardiovascular<br>Respiratory<br>Gastrointestinal<br>Mental health<br>Neurologic	5-month postdischarge follow-up	NR	4<br>Murad et al.

Study	Study Type<br><br>Country	N (total)<br>Gender (%M or F)	MIS<br>clinical features/ symptoms	Age<br>Mean ±SE/ Median (IQR) (years)	Comorbidities (before COVID)	COVID-19<br>Date and duration<br><br>Severity	*Diagnosis	Blood markers	Management/ Treatment	Any other complications	How long follow up<br><br>Outcome	vaccination status	QA
										Infectious disease<br>Inflammatory<br>Systemic			
		COVID-19 (372)<br>47.3%	Respiratory<br>Neurologic<br>Mental health reasons	11.10 (5.82)<br>Median NR	1 or more CDC covid risk mean and SE: 45.4%	NR							
		MIS-C (183)<br>71.6%	Cardiovascular<br>Gastrointestinal<br>inflammatory conditions	9.12 (4.62)<br>Median NR	1 or more CDC covid risk mean and SE: 25.1%	NR							
He et al. [61]	Retrospective single-center Longitudinal assessment of patients<br>USA	N=22<br>59% M	Acute/ subacute/ chronic phase Patients based on parasternal short axis at the papillary muscle at the three phases of the disease	7.5 (5-13.5)	NR	NR	Acute / subacute (14-42 days after)/ chronic phase (>42 days)	Troponin T (0.00-0.13 ng/mL)	Aspirin<br>IVIG<br>Steroids<br>Anakinra<br>PICU admission<br>Vasopressor<br>Inotrope support<br>Mechanical ventilation	NR	NR	NR	6<br>NOS
			Cardiovascular dysfunction					BNP (5566 pg/mL)					
			Myocardial injury					CRP (199 mg/L)					
			GI symptoms					Ferritin (422 ng/mL)					
			Cardiogenic shock					Creatinine (0.49 mg/dL)					
Su et al. [41]	Case presentation	N= 1<br>100 % F	Oxygen pressure about 66 mg	78 years	NR	About 2-3 weeks	Diagnosed 19 days into COVID	IL-6 100 pg/mL	Nirmatrelvir/ritonavir	Shortness of breath	NR	NR	6<br>Murad et al.
			Pneumonia			severe		IL-8 100 pg/mL	Empirical antibiotics	Chest distress			

Study	Study Type<br><br>Country	N (total)<br>Gender (%M or F)	MIS clinical features/ symptoms	Age Mean ±SE/ Median (IQR) (years)	Comorbidities (before COVID)	COVID-19 Date and duration<br><br>Severity	*Diagnosis	Blood markers	Management/ Treatment	Any other complications	How long follow up<br><br>Outcome	vaccination status	QA
			Fi02 80%					IL-2	Moxifloxacin	Generalized fatigue			
			Hypotension					IL-12P 70	Methylprednisolone	Allotriophagy			
			Diarrhea					IFN-y	Meropenem	Cough			
			Multisystem injury including myocardial injury					Platelet count dropped sharply	IVIG				
			Thrombus right lower extremity					Change of blood cell count	Renal replacement therapy				
Das et al. [27]	Single-center retrospective study India	N=9<br>56% M	Diarrhea	Mean= 40 years SD= 13 years	Obesity (BMI 42)	Patients had infection or contact history with a mean of 36.8 days back	4 patients had mild COVID infection	High CRP	IV hydrocortisone (50-100 mg q6h-q8h)	6 patients recovered completely	NR	A female unvaccinated patient was treated with cefoperazone-sulbactam and doxycycline and did not require steroids	7 NOS
			Vomiting	18-21 years	HTN		The others had contact history	High D-dimer	Antibiotics			She was given tablet colchicine 0.5mg twice daily for 3 days	
			Abdominal pain and discomfort		Hypothyroid			High ferritin					
			Odynophagia and cervical lymphadenopathy		DM			High PCT					
			Cardiovascular (ventricular		Coronary artery disease			Neutrophilic leukocytosis					

Study	Study Type<br><br>Country	N (total)<br>Gender<br>(%M or F)	MIS<br>clinical features/<br>symptoms	Age<br>Mean ±SE/<br>Median<br>(IQR)<br>(years)	Comorbidities (before COVID)	COVID-19<br>Date and duration<br><br>Severity	*Diagnosis	Blood markers	Management/<br>Treatment	Any other complications	How long follow up<br><br>Outcome	vaccination status	QA
			dysfunction), respiratory, renal and hepatic involvements										
Baker et al. [18]	Case presentation	N=1<br>100% M	Localized peritonism in the right iliac fossa	32 years	Obesity	2 months earlier severity not mentioned	After infection (after over 2 months from having COVID)	Raised inflammatory markers	IV antibiotics were administered	NR	NR	NR	5<br>Murad et al.
			Lymphadenopathy		Ipregnancy intrahepatic cholestasis			ALP 162U/L	Tested for HIV, TB, EBV and CMV				
			Fat stranding					GGT 60 U/L	Follow-up TTE				
			Abdominal pain					Bilirubin 27 micromol/L	IVIG and corticosteroid therapy				
			Fever					Troponin 7800 ng/mL	Oral prednisolone				
Mejias et al. [66]	Electronic Cohort Study<br>United States of America	N=1025349<br>Gender=NR	NR	SARS-CoV-2 Infected 8.7 years<br>SARS-CoV-2 PCR 8.7 years<br>SARS-CoV-2 Serology 9.5 years	Chronic conditions (unspecified)	Between March 2020 and April 2022<br>Date and Duration NR	NR	Elevated inflammatory markers	Vaccination	Post-acute sequalae<br>Post acute manifestation	NR	Children who were unvaccinated were excluded from this study	9<br>NOS
Keka-Sylaj et al. [63]	Case Report<br>Kosovo,	N=1<br>100% M	Lethargic<br>Irritable<br>Loss of appetite	Age=5<br>Mean/SE=NR	Nasopharyngeal inflammation	December 2021<br>Duration=NR<br>Severe	Initial symptoms appear	CRP 156.8 mg/L	AntibioticSCeftriax-one (80	Photophobia<br>Multi organ dysfunction	Every 3 months for one year	NR	5<br>Murad et al.

Study	Study Type<br><br>Country	N (total)<br>Gender<br>(%M or F)	MIS<br>clinical features/<br>symptoms	Age<br>Mean ±SE/<br>Median<br>(IQR)<br>(years)	Comorbidities (before COVID)	COVID-19<br>Date and duration<br><br>Severity	*Diagnosis	Blood markers	Management/<br>Treatment	Any other complications	How long follow up<br><br>Outcome	vaccination status	QA
	Albanian Child		Bilateral<br>Nonpurulent conjunctivitis<br>Oral mucosal lesions<br>dry lips<br>“strawberry” red tongue<br>Fever<br>Oropharyngeal hyperemia<br>Headache<br>Abdominal pain<br>Vomiting<br>Swollen red eyelids<br>Swelling in palms and soles of feet		Hyperinflammatory syndrome<br>Obesity<br>Urinary tract infection<br>Gastroenteritis		ed 3 weeks prior<br>Diagnosed January 2021		mg/kg/day)<br>Vancomycin (40 mg/kg/day)<br>Methylprednisolone (1 mg/kg/day)<br>IVIG (1 g/kg)<br>Anticoagulation therapy with enoxaparin	Hyperinflammatory syndrome			
								PCT 13.84 ng/mL					
								ESR 100 mm/hour					
								Urea 15.81					
								Creat 114 umol/L					
								Albumin 30.6 g/L					
								ALT 36 U/L					
								AST 39 U/L					
								Amylase 37 U/L A					
								ALP 179 U/L					
								GGT 74 U/L					
								CK 20 U/L					
								LDH 460 U/L					
Al-Mashdali et al. [16]	Case Presentation<br>Qatar	N=1<br>M%=100%	Fever<br>Diarrhea<br>Vomiting	Age=21<br>Mean=NR<br>SE=NR	None before COVID<br>No Chronic Illnesses	Date=NR<br>Duration=Few Days<br>Mild COVID-19	3 weeks after	CRP 156.4 mg/L	Ceftriaxone<br>Piperacillin-tazobactam<br>Meropenem	Developed Bell’s Palsy after MIS (Right -side	3 days	NR	6<br>Murad et al.

Study	Study Type<br><br>Country	N (total)<br>Gender<br>(%M or F)	MIS<br>clinical features/<br>symptoms	Age<br>Mean ±SE/<br>Median<br>(IQR)<br>(years)	Comorbidities (before COVID)	COVID-19<br>Date and duration<br><br>Severity	*Diagnosis	Blood markers	Management/<br>Treatment	Any other complications	How long follow up<br><br>Outcome	vaccination status	QA
			Severe<br>Abdominal Pain<br>bilateral non-purulent<br>conjunctivitis<br>Enlarged Tender<br>Left cervical<br>lymph node<br>hypotension<br>Tachypneic				COVID-19<br>Five<br>days		Linezolid<br>Intubation to relieve respiratory distress	facial weakness)			
								WBC 4.7 × 10^3/ul					
								Hgb 11.5 g/dl					
								Platelet 111 × 10^3/ul					
								Urea 3.6 mmol/L					
								Creatinine 90 umol/L					
								ALT 57 U/L					
								AST 92 U/L					
								PCT 0.30 ng/ml					
Campanella et al. [55]	Case Report<br>Italy	N=1<br>M%=100%	Abdominal pain<br>Nausea<br>Fever<br>Tachycardia<br>Palmar rash<br>Petechiae	Age=9 years<br>Mean=NR	NR	6 days<br>Date=NR<br>Severity NR	2 days	Elevated CRP	Appendectomy<br>IVIG<br>IV Steroid Therapy	Blumberg's Sign and Rovsing's Sign were positive	None Mentioned	NR	5<br>Murad et al.
								Elevated PCT					
Mazumder et al. [37]	Case Report	N=1<br><br>100% M	Decreased Sensation in both feet<br>Weakness in both lower limbs<br>Clumsiness of both hands<br>Decreased Urine Output<br>Nausea	Age=61 years<br>Mean=NR	HTN<br>Hypothyrodism<br>Coronary artery disease<br>Percutaneous transluminal coronary angioplasty (PCTA)	Date NR<br>3 days<br>Mild COVID-19	One week afterwards	Hgb of 8 g/dL	Antibiotics<br>Intermittent HD<br>Blood and fresh frozen plasma<br>IVIG<br>Selective Arterial	Ileal Bleed<br>Lower gastrointestinal bleed	3 weeks	Unvaccinated against COVID-19 at time of hospital visit	5<br>Murad et al.

Study	Study Type<br><br>Country	N (total)<br>Gender<br>(%M or F)	MIS<br>clinical features/<br>symptoms	Age<br>Mean ±SE/<br>Median<br>(IQR)<br>(years)	Comorbidities (before COVID)	COVID-19<br>Date and duration<br><br>Severity	*Diagnosis	Blood markers	Management/<br>Treatment	Any other complications	How long follow up<br><br>Outcome	vaccination status	QA
			Vomitting<br>Severe pain and swelling in both thighs<br>Aferbile						Embolization				
								WBC 15,300/mm^3					
								10% lymphocytes					
								platelet count 2.14 L/cmm					
								BUN 67.23 mg/dL serum					
								creatinine 8.02 mg/dL					
								sodium 127 mmol/L					
								potassium 5.43 mmol/L					
								AST 862 U/L					
								ALT 490 U/L					
								troponin T 389 pg/mL					
								BNP 10,800 pg/mL					
								PCT 0.968 ng/mL					
								CRP 55.8 mg/L					
								ferritin 1,100 ng/mL					
								LDH 7,250 U/L					
								CK 55,920 U/L					
								CK-MB 34 U/L					
								TSH 30.53 mIU/L					

Study	Study Type<br><br>Country	N (total)<br>Gender<br>(%M or F)	MIS<br>clinical features/<br>symptoms	Age<br>Mean ±SE/<br>Median<br>(IQR)<br>(years)	Comorbidities (before COVID)	COVID-19<br>Date and duration<br><br>Severity	*Diagnosis	Blood markers	Management/<br>Treatment	Any other complications	How long follow up<br><br>Outcome	vaccination status	QA
								D-dimer 5.57 mg/mL					
Aziz et al. [51]	Cohort<br>Non-Interventional Descriptive Study<br>Country NR	N=41<br>M%=73%	Impaired LVEF<br>Coronary Artery Involvement<br>Pericardial Effusion	Median Age= 7 years<br>Range= 0.2-16 years<br>Mean and SE=NR	Kawasaki Disease	NR	NR patients admitted to the hospitals between May 2020 and April 2021	NR	NR	Impaired cardiac function	1, 3, 6, 12, and 16 months	NR	6<br>NOS
Ajmi et al. [47]	Case Report<br>Italy<br>Tunisian Girl	N=1<br>100% F	Fever<br>Irritability<br>Bilateral Submandibular Adenopathy<br>Lymphopenia<br>Diarrhea<br>Tachycardia	12 years old<br>Age Mean and<br>SE=NR	NR	NR<br>Detection 15 days prior hospital admission<br>Severity NR	NR	CRP 359 mg/l	Norepinephrine<br>Broad-Spectrum antibiotics-imipenem, vancomycin and amikacin<br>IV antibiotics-cefotaxime and gentamycin<br>Dobutamine<br>Hydrocortisone<br>IVIG	Hypotension<br>Pelvic ectopic right kiidney<br>Pelvic Peritoneal Effusion<br>Regardless of the medication, the patient had a fatal outcome	NR	Positive RT-PCR 15 days prior hospital admission	4<br>Murad et al.
								SGOT 491 UI/l					
								SGPT 184 UI/l					
								BUN 15 mmol/l					

Study	Study Type<br><br>Country	N (total)<br>Gender<br>(%M or F)	MIS<br>clinical features/<br>symptoms	Age<br>Mean ±SE/<br>Median<br>(IQR)<br>(years)	Comorbidities (before COVID)	COVID-19<br>Date and duration<br><br>Severity	*Diagnosis	Blood markers	Management/<br>Treatment	Any other complications	How long follow up<br><br>Outcome	vaccination status	QA
								Creatinine 360 µmol/l					
								Sodium 129 mmol/l					
								Potassium 2.6 mmol/l					
								Leukocytes 15.95 × 10^9/l					
								ALC 0.6 × 10^9/l					
								Platelets 110 × 10^9/l					
Sai et al. [73]	Prospective Observational Study<br>India	N=78<br>M%=34.6 %	Rash (p=0.03)<br>Conjunctival Congestion (p=0.021)<br>Cough (p=.567)<br>Cold (p=0.058)<br>Vomiting (p=0.099)<br>Loose Stool (p=0.608)<br>Abdominal pain (p=0.279)<br>Lethargy (p=0.099)<br>Poor Oral Intake (p=0.223)	Mean and SE =NR<br>Patients less than 1 years old=20.5%<br>Patients 1-5 years old= 35.9%<br>Patients 6-12 years old=43.6%	NR	NR<br>Patients taken from teaching hospital in a period of 12 months<br>December 2020-November 2021	NR	D-Dimer 34.6%	Fluid Bolus=74.4%<br>Inotropic Support= 35.9%<br>Renal Replacement =1.2%<br>Ventilation=7.6%<br>Steroid=70.5 %<br>IVIg=94.9%	NR	NR<br>77 children survived<br>1 Child died	Girls were affected more than boys= (M: F ratio of 1:1.9)	3<br>NOS
								PT-INR 10.2%					
								aPTT 29.5%					
								Leukocytosis 44.9%					
								Thrombocytopenia 20.5%					
Aeschlimann et al. [46]	Multicenter Internati	N=111<br>62% M	Myocarditis (20)<br>Myocardial necrosis, fibrosis	Median Age=10.0 years	Bronchiolitis<br>Asthma (16)<br>Autism (2)	Consecutive Patients Collected	Average 5 days	Hgb 11.2 g/L	NR	NR	55 days	NR	8<br>NOS

Study	Study Type<br><br>Country	N (total)<br>Gender (%M or F)	MIS clinical features/ symptoms	Age Mean ±SE/ Median (IQR) (years)	Comorbidities (before COVID)	COVID-19 Date and duration<br><br>Severity	*Diagnosis	Blood markers	Management/ Treatment	Any other complications	How long follow up<br><br>Outcome	vaccination status	QA
	onal Center Approved by Ethics Board in Paris, France		Pericardial Effusion<br>Coronary Dilation<br>Fever<br>Respiratory Symptoms<br>Gastrointestinal Symptoms<br>Arrhythmias<br>Chest Pain<br>Dyspnea<br>Renal failure<br>Circulation failure	IQR=7.0-13.8	Overweight/obese (8)<br>Myocarditis (1)<br>Chronic tuberculosis (1)<br>Psoriasis (1)<br>Arterial HTN (1)<br>Systemic lupus (1)<br>Congenital adrenal hyperplasia (1)<br>Alopecia Areata (1)	In March 2020 to April 2021<br>COVID severity and duration NR							
								Leucocytes (/L) 18.1					
								Lymphocytes (L) 1.5					
								Platelets L 183					
								CRP mg/L 23.8					
								BNP ng/L 932					
								NT pro-BNP ng/L 4818					
								TnT ng/L 110					
								TnT peak ng/L 205					
Pettinato et al. [40]	Case Report	N=1<br>M%=0	Fever<br>Weakness	43 year old female	NR	Date and Duration NR	Soon after,	Elevated Ferritin	Corticosteroids	Hypothyroidism	2 months later,	Not vaccinated	4 Murad et

Study	Study Type<br><br>Country	N (total)<br>Gender<br>(%M or F)	MIS<br>clinical features/<br>symptoms	Age<br>Mean ±SE/<br>Median<br>(IQR)<br>(years)	Comorbidities (before COVID)	COVID-19<br>Date and duration<br><br>Severity	*Diagnosis	Blood markers	Management/<br>Treatment	Any other complications	How long follow up<br><br>Outcome	vaccination status	QA
	United States of America		Dysphagia<br>Vomiting<br>Diarrhea<br>Maculopapular Rash<br>Chest tightness<br>Palpitations<br>Hypotensive			COVID<br>Entered the hospital five days after fever developed	Exact time:<br>NR		Levothyroxine<br>Nitroglycerin<br>Aspirin<br>Heparin<br>Morphine<br>Ondansetron	Acute Renal Failure<br>Lactic Acidosis	revisited for chest tightness	against SARS-CoV-2 at time of presentation	al.
								Elevated CRP					
								Elevated D-Dimer					
								Elevated Troponin					
Basu et al. [54]	Descriptive study<br>India	N=10<br>80% M	Fever<br>Rash<br>Mucositis<br>Conjunctivitis<br>Vomiting<br>Diarrhoea<br>Abdominal pain<br>Shock<br>Cardiac injury	6<br>(IQR: 4-10)	NR	NR	2-4 weeks post infection	High CRP	IVIG<br>Glucocorticoids<br>IL-1or IL-6 inhibitors	Organ involvement<br>Shock<br>Cardiac injury<br>Gastrointestinal issues<br>Hyperinflammatory response<br>Myocarditis	NR	NR	6<br>NOS
								Lymphopenia					
Budhiraja et al. [23]	Case Report<br>India	N=1<br>100% M	Fever<br>Polyarthralgia<br>Palpitation<br>Breathless<br>Mouth soreness<br>Tachycaria<br>Conjunctivitis<br>Mucositis,<br>Erythematous blanching rash	44	NR	Last week of April 2021<br>Mild illness with breif duration of fever; Home managemnet	Mid June 2021,<br><br>Presented to hospital in first week of July 2021	CRP (mg/dl)<br>326	Methylprednisolone<br>LMWH (enoxaparin)<br><br>IVIG<br>Valsartan-Sacubitril,<br>Carvedilol<br>Diuretic	Myocarditis, global hypokinesia with reduced LVEF	One week follow up	NR	5<br>Murad et al.
								IL-6 (pg/ml)<br>371.3					
								Serum Ferritin					

Study	Study Type<br><br>Country	N (total)<br>Gender<br>(%M or F)	MIS<br>clinical features/<br>symptoms	Age<br>Mean ±SE/<br>Median<br>(IQR)<br>(years)	Comorbidities (before COVID)	COVID-19<br>Date and duration<br><br>Severity	*Diagnosis	Blood markers	Management/<br>Treatment	Any other complications	How long follow up<br><br>Outcome	vaccination status	QA
								(ng/ml<br>1201.9					
Loncharich et al. [36]	Retrospective<br>Case series<br>US	8<br>(4 Pediatric, 4 adult)<br>P: 50% M<br>A: 75% M	Fever<br>Rash<br>Conjunctivitis<br>Reduced feeding<br>Myalgias<br>Arthralgia<br>Sore throat<br>Chest pain<br>Nausea<br>Vommiting<br>Abdominal pain<br>Headache<br>Syncope<br>Dyspnoea	P: 17 months-15 years<br>A: 21-41 years	Asthma<br>Anxiety	March 2020 -June 2021<br><br>1-16 weeks<br><br>Asymptomatic to acute respiratory failure	From exposure till 16 weeks	CRP	IVIG, Pulse-dose steroids<br>anakinra<br>aspirin<br>enoxaparin	Coronary artery ectasia<br>Myocarditis<br>Diastolic dysfunction<br>Reduced systolic function<br>Acute hypoxic respiratory failure	1, 3 and 6 months	NR	5<br>Murad et al.
								Ferritin					
								ESR					
								Leucocytosis					
								Anaemia					
								Troponin					
								BNP					
AkaslanKara et al. [48]	Case Report<br>Turkey	N=1<br>100% M	High fever<br>Tachycardia<br>Chest pain	13	Thrombus in tricuspid valve	2.5 months before admission.<br><br>Hospitalization	3 weeks after admission	Leucocyte<br>10,640 cells/mm^3	IVIG (2g/kg)<br><br>acetylsalicylic acid<br>(5mg/kg)	Thrombus in multiple locations<br>Thorax bacterial consolidation<br>Pneumonia<br>PE	NR	NR	5<br>Murad et al.
								Lymphocyte<br>4,600 cells/mm^3					
								Platelets<br>388,000 cells/mm^3					

Study	Study Type<br><br>Country	N (total)<br>Gender<br>(%M or F)	MIS<br>clinical features/<br>symptoms	Age<br>Mean ±SE/<br>Median<br>(IQR)<br>(years)	Comorbidities (before COVID)	COVID-19<br>Date and duration<br><br>Severity	*Diagnosis	Blood markers	Management/<br>Treatment	Any other complications	How long follow up<br><br>Outcome	vaccination status	QA
								Hgb 11.1 g/dL					
								CRP 0.2 mg/dL					
								ESR 27 mm/h					
								D-dimer 244 ng/mL					
								fibrinogen levels 265 mg/dL					
								Mean Leucocyte count (10^9/L) 13.6 ± 6.9					
								Mean Neutrophil count % 76.5 ± 15.0					
								Mean Lymphocyte count % 18.8 ± 12.8					
								ALC (10^9/L) 2.13±0.95					
								Mean Platelet count (10^9/L) 238.5 ± 206.0					
Tamarkee et al. [77]	Case Report UAE	1<br>Female	Fever<br><br>Throat pain<br><br>Epigastric pain<br><br>Maculopapular erythematous rash<br><br>Vomiting	4	NR	NR	Family history of COVID 19 before two weeks.<br><br>Tested positive for COVI	WBC 6.23 × 103 /mm^3	IVIG (2g/kg)<br>IV ceftriaxone<br>aspirin(300mg/kg/day), discharged on aspirin (25mg/kg for 4 weeks)	Abnormal ECG<br>Mild hepatomegal<br>Multiple enlarged bilateral cervical lymph nodes	2 weeks, 1 month	NR	5<br>Murad et al.

Study	Study Type<br><br>Country	N (total)<br>Gender<br>(%M or F)	MIS<br>clinical features/<br>symptoms	Age<br>Mean ±SE/<br>Median<br>(IQR)<br>(years)	Comorbidities (before COVID)	COVID-19<br>Date and duration<br><br>Severity	*Diagnosis	Blood markers	Management/<br>Treatment	Any other complications	How long follow up<br><br>Outcome	vaccination status	QA
			Conjunctivitis<br><br>Strawberry tongue<br><br>Cervical lymph nodes congested<br><br>Throat with hyperemic tonsils				D infection-specific IGM, IGG						
								RBC 4.64 million/mm^3					
								Hgb 10.8 g/dL					
								Platelet 0.93 × 10^5/mm^3					
								CRP 74.6 mg/dL					
								SODIUM 136 mmol/L					
								POTASSIUM 3.8 mmol/L					
Gillrie et al. [30]	Case Report<br>Canada	N=1<br>100% M	Fever<br>Abdominal pain<br>Diarrhoea<br>Macular blanching skin rash<br>Bilateral conjunctivitis<br>Sore throat<br>Chest pressure<br>Hypotensive	38	NR	Around 25 days before symptoms.<br>Mild upper respiratory tract infection	Two weeks post recovery, PCR positive (Ct-value >30), SARS-CoV-2 serology (spike RBD and N proteins)	Elevated inflammatory markers	Aspirin<br>Enoxaparin<br>Broad-spectrum Antibiotics<br>Oral dexamethasone	Severe biventricular heart failure<br><br>Myocarditis<br><br>Potential acute coronary syndrome<br><br>Renal failure<br><br>Diarrhoea<br><br>Abdominal pain	30 days post discharge	Unvaccinated	

Study	Study Type<br><br>Country	N (total)<br>Gender<br>(%M or F)	MIS<br>clinical features/<br>symptoms	Age<br>Mean ±SE/<br>Median<br>(IQR)<br>(years)	Comorbidities (before COVID)	COVID-19<br>Date and duration<br><br>Severity	*Diagnosis	Blood markers	Management/<br>Treatment	Any other complications	How long follow up<br><br>Outcome	vaccination status	QA
							strongly positive						
								Elevated renal makers					
								Elevated hepatic markers					
								Elevated coagulation markers					
								Elevated CRP					
								Elevated BNP					
								Elevated troponin					
								Elevated D-Dimer					
Barth et al. [19]	Case studies, USA	N=3<br>100% M	Acute onset cardiogenic shock<br>Glucose intolerance<br>Unmasking of autoimmune disease<br>Sepsis	31	Case 1 person had Febrile respiratory illness, Obstructive sleep apnea, Anxiety and Degenerative disc disease<br><br>Case 2 and 3 had no comorbidities before COVID.	Case 1 had COVID-19 two months prior to admission<br><br>Case 2 and 3 positive for COVID-19 upon admission.	Diagnosed 2 months after infection for Case 1<br><br>During infection for Case 2 and 3	Case 1:<br>Troponin<br>4.24 µg/	Standard of care interventions plus immunomodulatory therapy that included IVIG Corticosteroids and in two cases Anakinra	Worsening dyspnea, cough	Case 1 - 14 days<br>Case 2 - 24 days<br>Case 3 - 35 days	Case 1 and 2 were unvaccinated.<br>Case 3 had received an initial dose of SARS-CoV-2 mRNA vaccine 6 weeks prior	5<br>Murad et al.
								BNP<br>1618 pg./mL					
								CRP<br>443 mg/L					

Study	Study Type<br><br>Country	N (total)<br>Gender<br>(%M or F)	MIS<br>clinical features/<br>symptoms	Age<br>Mean ±SE/<br>Median<br>(IQR)<br>(years)	Comorbidities (before COVID)	COVID-19<br>Date and duration<br><br>Severity	*Diagnosis	Blood markers	Management/<br>Treatment	Any other complications	How long follow up<br><br>Outcome	vaccination status	QA
								WBC<br>25.9×109/L					
								Ferritin<br>5,056 μg/L					
								glucose<br>382 mg/dL					
								Case 2:<br>Troponin<br>50 μg/L					
								BNP<br>300 pg./mL					
								CRP 232 mg/					
								Ferritin<br>15,000 μg/L					
								Blood<br>glucose<br>1,959 mg/dL					
								HbA1c > 14<br>%,					

Study	Study Type<br><br>Country	N (total)<br>Gender<br>(%M or F)	MIS<br>clinical features/<br>symptoms	Age<br>Mean ±SE/<br>Median<br>(IQR)<br>(years)	Comorbidities (before COVID)	COVID-19<br>Date and duration<br><br>Severity	*Diagnosis	Blood markers	Management/<br>Treatment	Any other complications	How long follow up<br><br>Outcome	vaccination status	QA
								pH < 6.78					
								Case 3:<br>Troponin<br>4.55 µg/					
								Pro-BNP<br>34,645 pg./dL					
								CRP<br>362.7 mg/L					
								WBC<br>31.8×109/L					
								Ferritin<br>8,219 µg/L					
Nawfal et al. [38]	Case study, Lebanon	N=1<br>Female<br>(100%)	New-onset refractory status epilepticus (NORSE)<br>Unremitting nausea and abdominal pain associated with a high-grade fever<br>MRI showed symmetric T2-signal increase involving both orbitofrontal lobes, insulae, and hippocampi	21	NR	Mild COVID-19 infection 5 weeks prior to presentation	5 weeks after COVID infection	HCt 29%	IV methylprednisolone<br>IVIG	Generalized fatigue	3 months	Not vaccinated	4<br>Murad et al.
								ESR 79 mm/h					

Study	Study Type<br><br>Country	N (total) Gender (%M or F)	MIS clinical features/ symptoms	Age Mean ±SE/ Median (IQR) (years)	Comorbidities (before COVID)	COVID-19 Date and duration<br><br>Severity	*Diagnosis	Blood markers	Management/ Treatment	Any other complications	How long follow up<br><br>Outcome	vaccination status	QA
								CRP 130 mg/L					
								Ferritin 380 ng/mL					
								IL-6: 25 pg/mL					
								D-Dimer 1900 ng/mL					
								COVID IgG 1456 AU/mL					
Zahornacky et al. [43]	Case study<br><br>Slovakia	N=1<br>100% M	SOFA score 5<br>Fevers up to 40 °C persisted<br>Dyspnea<br>Hypotension<br>Tachycardia<br>Maculopapular exanthema on chest<br>Eyelid edema<br>Nonpurulent conjunctivitis	22 years	none	Ct cycle of 33.54	22 days	CD4 + 0.10 × 10^9/L,	IVIG 2 g/kg (130 g in total)<br><br>Methylprednisolone 1 mg/kg (5 days)<br><br>Piperacillin/tazobactam with gentamicin<br><br>Anakinra/ infliximab<br>Cochicine+beta blocker	Pleural, Pericardial effusions<br>Pneumonia	Discharged after 22 days<br><br>Follow up duration not mentioned	Vaccinated	4<br>Murad et al.

Study	Study Type<br><br>Country	N (total)<br>Gender<br>(%M or F)	MIS<br>clinical features/<br>symptoms	Age<br>Mean ±SE/<br>Median<br>(IQR)<br>(years)	Comorbidities (before COVID)	COVID-19<br>Date and duration<br><br>Severity	*Diagnosis	Blood markers	Management/<br>Treatment	Any other complications	How long follow up<br><br>Outcome	vaccination status	QA
								CD3 + 0.14 × 10^9/L					
								CD8 + 0.04 × 10^9/L					
								natural killers 0.02 × 10^9/L					
								SPO2 92					
								High CRP					
								High IL-6					
								High PCT					
								High troponin,					
								High proBNP					
Goicea et al. [60]	Observational analysis of a group of pediatric patients, Romania	N = 18<br>39% M	Abdominal pains<br>Vomiting<br>Diarrhea<br>Exanthema<br>Headache<br>Agitation<br>Sleepiness<br>Myalgia<br>Meningits<br>Respiratory manifestation	5 (IQR 3-8.75)	NR	7 patients had COVID-19 in last 2-6 weeks	For 7 patients, diagnosed 2-6 weeks following COVID	Platelets ($x10^3/\mu L$)<br>150 (108.5-249.7)	NR	Cough<br>Headache<br>Coagulopathy<br>Jaundice	31st March to 31st December, 2020	None of the patients were vaccinated	6<br>NOS

Study	Study Type<br><br>Country	N (total) Gender (%M or F)	MIS clinical features/ symptoms	Age Mean ±SE/ Median (IQR) (years)	Comorbidities (before COVID)	COVID-19 Date and duration<br><br>Severity	*Diagnosis	Blood markers	Management/ Treatment	Any other complications	How long follow up<br><br>Outcome	vaccination status	QA
							infection						
								Neutrophil-to-lymphocyte ratio 7.06 (4.78-11.32)					
								Platelet-to-lymphocyte ratio 103.6 (78.92-163.7)					
								CRP (mg/L) 144.4 (53.92-212.7)					
								Ferritin (ng/mL) 710.4 (491-1,527)					
								D-dimer (ng/mL) 910 (577.6-1,825)					
								Troponin (pg/mL) 9 (4.82-20.2)					
								proBNP (pg/mL) 1,206 (1,123-1,742)					

Study	Study Type<br><br>Country	N (total)<br>Gender<br>(%M or F)	MIS<br>clinical features/ symptoms	Age<br>Mean ±SE/ Median (IQR) (years)	Comorbidities (before COVID)	COVID-19<br>Date and duration<br><br>Severity	*Diagnosis	Blood markers	Management/ Treatment	Any other complications	How long follow up<br><br>Outcome	vaccination status	QA
Sahu et al. [72]	Case Report, India	Case 1:<br>N=1<br>0% M	Abdominal pain for 8 days, diffuse abdominal tenderness	10	NR	Patient tested positive when for anti-SARS COV	After recovery from COVID	CRP (249 mg/L)	Empirical antibiotics<br><br>Paracetamol<br><br>IV fluid	NR	NR	NR	3<br>Murad et al.
			Loss of motion (5 days)					D-dimer 5.06 µg/mL	IVIG therapy				
			Pericardial effusion					LDH 281 u/L					
			US suggested perforated appendix with lump formation.					Ferritin 213 ng/mL					
								NT Pro BNP 174 pg/mL					
		Case 2:<br>N=1<br>100% M	Abdominal pain, diffuse abdominal tenderness, vomiting (2 days)<br><br>Abnormal liver function	6		Patient tests positive for anti-SARS COV	After recovery from COVID	Ferritin 680 ng/mL (7-140 ng/mL)	IVIG therapy<br><br>laparotomy with ileostomy				
			Dehydration					D-dimer 8.5 mcg/mL					
			High grade fever					CRP 129.4 mg/L					

Study	Study Type<br><br>Country	N (total)<br>Gender<br>(%M or F)	MIS<br>clinical features/<br>symptoms	Age<br>Mean ±SE/<br>Median<br>(IQR)<br>(years)	Comorbidities (before COVID)	COVID-19<br>Date and duration<br><br>Severity	*Diagnosis	Blood markers	Management/<br>Treatment	Any other complications	How long follow up<br><br>Outcome	vaccination status	QA
			Dilated bowel loops with multiple air-fluid levels										
			Ileal perforation										
Benli et al. [20]	A case series studies in Turkey	N=6<br>67% M	All patients were asymptomatic for a period after COVID	Mean is 43.3 years range: 23-56	Allergic rhinitis	All patients had history of mild COVID in last 2-8 weeks	Period between COVID and MIS-A diagnosis:29, 10, 24, 29, 50, 12 days	CRP 111-412/µL	⅚ patients received corticosteroids	All patients had positive SARS-CoV-2 antibodies	NR	All patients were unvaccinated against SARS-CoV 2	5<br>Murad et al.
			All patients presented with fever and severe fatigue		Nephrolithiasis			Ferritin 426.3-2748 ng/mL	IVIG for 2 severe cases	Abnormal echocardiography (83.3%)			
			None had respiratory problems		Inactive hepatitis B Type 2 DM, HTN			D-dimer 1.4-2900 µg/L	LMWH 40mg/day				
			Severe hypotension (83.3%)					ProBNP 6.831-1006 PG/mL	Anakinra				
			5 patients abnormal cardiography					PCT 0.73-11.62 ng/mL	Antibiotic treatment prophylactic				

Study	Study Type<br><br>Country	N (total)<br>Gender (%M or F)	MIS clinical features/ symptoms	Age Mean ±SE/ Median (IQR) (years)	Comorbidities (before COVID)	COVID-19 Date and duration<br><br>Severity	*Diagnosis	Blood markers	Management/ Treatment	Any other complications	How long follow up<br><br>Outcome	vaccination status	QA
			1 patient mucocutaneous findings					Lymphocyte 300-800µl					
			Lymphopenia					Neutrophil 2350-25600/µl					
			Mild to moderate thrombocytopenia					Platelets75000-622000/µl					
								Troponin 3.54-540.5 pg/mL					
Bari et al. [53]	Descriptive cross-sectional study Pakistan	66<br>57.6% M	Not mentioned	7.9 ± 4.2 years for total population MIS-C only 7.5 ± 3.3 years	Chronic kidney disease Congenital heart disease Surgical cases	Mar-Sep 2020 Asymptomatic (5) Mild-mod (38) Severe-critical (23)	NA	No markers, only mentions blood groups	Not mentioned	Resp support None 40 O2 23 Mechanical Ventilation 3 ICU/HDU None 39 ICU 12 HDU 15	f/up not mentioned<br><br>Outcome Discharged 61 Expired 5		6 NOS
Herrera-Garcia et al. [31]	Case report Mexico	N=1<br><br>100% M	Presented to ED with 85% saturation 113bpm RR 28 breaths/min afebrile 80/40 mmHg MMRC 2-3 dyspnea weakness fatigue tiredness increasing chest pain chest tightness intolerance to movement	56 year old male	DM	Positive COVID-19 antigen test 1 month prior to admission with upper airway infection and dyspnea. Received steroids, oxygen, but little improvement and had intermittent hypoxia; after the month of treatment he had weakness, chest pain, and requed continuous O2 at 3L/min	30d post admission, after treatment, improvement within 24hr	Hgb<br><br>Day 0 12g/dL	Steroids<br><br>Triple antibiotic therapy after an echocardiogram found a vegitative image in the mitral valve<br><br>After 15-20 days, only partial relief	See management and clinical description	NA	Article in Spanish, possible translation errors; vaccination status not mentioned	4 Murad et al.

Study	Study Type<br><br>Country	N (total)<br>Gender (%M or F)	MIS clinical features/ symptoms	Age Mean ±SE/ Median (IQR) (years)	Comorbidities (before COVID)	COVID-19 Date and duration<br><br>Severity	*Diagnosis	Blood markers	Management/ Treatment	Any other complications	How long follow up<br><br>Outcome	vaccination status	QA
			Chest CT showed pneumonia of 40% of lung w/ ground glass and multi atelectasis<br><br>Echocardiogram showed myopericarditis<br><br>He had elevated CRP, ESR, ferritin, and D-dimer						of symptoms was documented with continued weakness, desaturation, and hypotension that needed vasoactive amines for 10-15 days<br><br>Patient was later withdrawn from medication b/c of dyspnea (unable to move from hospital bed)<br><br>In dyspnea events, treated with anti-inflammatory drugs and bronchodilators (nebulizations)<br><br>Later referred to rehab service for diaphragmatic weakness therapy and				

Study	Study Type<br><br>Country	N (total)<br>Gender (%M or F)	MIS clinical features/ symptoms	Age Mean ±SE/ Median (IQR) (years)	Comorbidities (before COVID)	COVID-19 Date and duration<br><br>Severity	*Diagnosis	Blood markers	Management/ Treatment	Any other complications	How long follow up<br><br>Outcome	vaccination status	QA
									respiratory exercises allowing him to stand upright without need for amines<br><br>3-5d later, patient had relief of symptoms w/ help of hyperproteic diet<br><br>CRP decreased and ferritin increased so baricitinib was described for “COVID-19 with sequelae and chronicity”<br><br>After 30d hospitalization, symptoms were not completely relieved (severe event of oppressive chest pain, dyspnea, profuse diaphoresis, hypotension, desaturation, fatigue, and				

Study	Study Type<br><br>Country	N (total)<br>Gender<br>(%M or F)	MIS<br>clinical features/<br>symptoms	Age<br>Mean ±SE/<br>Median<br>(IQR)<br>(years)	Comorbidities (before COVID)	COVID-19<br>Date and duration<br><br>Severity	*Diagnosis	Blood markers	Management/<br>Treatment	Any other complications	How long follow up<br><br>Outcome	vaccination status	QA
									intolerance to movement), new acute phase reactants were requested with elevated results<br><br>Based on this MIS-A was diagnosed and a course of IVIG (2g/kg) and steroids was started with improvement after 24 hours<br><br>Home treatment based on O2, steroids, baricitinib, and pulmonary rehab alongside high protein diet				
								Leukocytes<br><br>Day 0 6K/uL					
								D-dimer					

Study	Study Type<br><br>Country	N (total)<br>Gender (%M or F)	MIS clinical features/ symptoms	Age Mean ±SE/ Median (IQR) (years)	Comorbidities (before COVID)	COVID-19 Date and duration<br><br>Severity	*Diagnosis	Blood markers	Management/ Treatment	Any other complications	How long follow up<br><br>Outcome	vaccination status	QA
								Day 0 4ug/mL					
								ESR<br><br>Day 0:60mm/h					
								PCT<br><br>Day 0: 0.5ng/mL					
								BNP<br><br>Day 0: 50pg/mL					
Coll et al. [25]	Case report<br><br>America	N=1<br><br>100% M	Presented to ED with 1 week of fever, right-sided neck swelling, progressive shortness of breath<br><br>Had depressed left ventricular function with hemodynamic and cardiogenic shock<br><br>Febrile, 125bpm, 103/64mmHg, 33 breathsmin, 94% O2 on 4L nasal cannula	39 year old male	None	Diagnosed with COVID-19 8 weeks prior to admission; received no antiviral or immunomodultory therapies and recovered normally	MIS-A detected upon hospitalization based on administration of IVIG	Sodium<br><br>Admission (day 1) 117mmol/L	Transthoracic ECG demonstrated LVEF of 30% w/ global hypokinesis and severely reduced RV function w/ tricuspid annular plane systolic excursion of 0.92cm and inferior vena cava measuring 3cm w/o	See management and clinical description	Discharged 9 dys after admission on prolonge prednisone taper<br><br>Sustained recovery of LV function post-discharge	Vaccination status not mentioned;<br><br>CRP, hs-TnT, and IL6 data approximated from figure 2 in the paper	6 Murad et al.

Study	Study Type<br><br>Country	N (total)<br>Gender (%M or F)	MIS clinical features/ symptoms	Age Mean ±SE/ Median (IQR) (years)	Comorbidities (before COVID)	COVID-19 Date and duration<br><br>Severity	*Diagnosis	Blood markers	Management/ Treatment	Any other complications	How long follow up<br><br>Outcome	vaccination status	QA
			Regular HR w/o murmurs, rubs or gallops<br><br>ECG showed diffuse PR depression, 1mm ST-segment elevations in I, avL, V5, V6 w/o reciprocal depression<br><br>CXR showed moderate pulmonary edema						respirophasic variation<br><br>Right heart catheterization showed midly elevated right and severely elevated left-sided pressures w/ low-normal cardiac index<br><br>Diagnosed systemic hyperinflammatory as cause of biventricular failure so treated with methylprednisolone 1g daily for 3d and IVIG for 0.5mg/kg per day for 4 d<br><br>Patient symptoms rapdily improved over course of first 24 hours with rapid improvement of biomarkers				

Study	Study Type<br><br>Country	N (total)<br>Gender<br>(%M or F)	MIS<br>clinical features/<br>symptoms	Age<br>Mean ±SE/<br>Median<br>(IQR)<br>(years)	Comorbidities (before COVID)	COVID-19<br>Date and duration<br><br>Severity	*Diagnosis	Blood markers	Management/<br>Treatment	Any other complications	How long follow up<br><br>Outcome	vaccination status	QA
									Inotropic support with dobutamine discontinued on day 2 of treatment<br><br>ECG on day 3 showed normalization of biventricular function				
								CO2<br><br>Admission (day 1) 23mmol/L					
								BUN<br><br>Admission 10mg/dL					
								Creatinine<br><br>Admission 0.78mg/dL					
								WBC<br><br>Admission 16.50K/uL					

Study	Study Type<br><br>Country	N (total)<br>Gender<br>(%M or F)	MIS<br>clinical features/<br>symptoms	Age<br>Mean ±SE/<br>Median<br>(IQR)<br>(years)	Comorbidities (before COVID)	COVID-19<br>Date and duration<br><br>Severity	*Diagnosis	Blood markers	Management/<br>Treatment	Any other complications	How long follow up<br><br>Outcome	vaccination status	QA
								Hgb<br><br>Admission<br>12.7g/dL					
								Platelets<br><br>Admission<br>307K/uL					
								Lactic acid:<br><br>Admission<br>2.5mmol/L					
								BNP<br><br>Admission<br>31877pg/mL					
								CK-MB<br>Admission<br>7.0ng/mL					
								ESR<br><br>Admission<br>100mm/h					
								PT-INR<br>Admission<br>1.2					
								PCT<br><br>Admission<br>0.40ng-mL					

Study	Study Type<br><br>Country	N (total)<br>Gender<br>(%M or F)	MIS<br>clinical features/ symptoms	Age<br>Mean ±SE/ Median (IQR) (years)	Comorbidities (before COVID)	COVID-19<br>Date and duration<br><br>Severity	*Diagnosis	Blood markers	Management/ Treatment	Any other complications	How long follow up<br><br>Outcome	vaccination status	QA
								SpO2 Admission 64%					
								hsTropT Day 1: 580ng/L					
								CRP<br><br>Day 1 280mg/L					
								IL-6<br><br>Day 1 180pg/mL					
AbiNassif et al. [44]	Case report Lebanon	N= 1<br><br>100% M	1-week history of highgrade fever (40°C) spiking every 5–6hours poorly responding to antipyretics<br>Chills<br>Myalgia<br>Severe headache<br>Tired and toxic and moderately dehydrated<br>Bilateral non-purulent bulbar (limbus-sparing) Conjunctivitis<br>Cracked, dry lips<br>Strawberry-red tongue with hyperemic oropharynx<br>Faint macular rash over the	8 year old	Not mentioned	1 month prior to presentaion<br>Mild symptoms	History of contact with known COVID-19 cases with no documented COVID-19 infection and mild symptoms developed 1 month prior to	WBC 10.2 cells ×10^9	Noninvasive respiratory support<br><br>Fluid resuscitationand was started on vasopressors (epinephrine and milrinone) for cardiac support<br><br>Broad-spectrum antibiotics were empirically initiated (IV	Cardiac function, moderate-to-severe mitral valve regurgitation<br>Moderate tricuspid regurgitation with an estimated right ventricular systolic pressure half systemic<br>Trace aortic regurgitation<br>Bilateral small pleural effusions<br>Distended inferior vena cava and	Admission on oral steroids, fursemide, captopril and lowdose acetysalicylic acid (aspirin)		6<br>Murad et al.

Study	Study Type<br><br>Country	N (total)<br>Gender<br>(%M or F)	MIS<br>clinical features/<br>symptoms	Age<br>Mean ±SE/<br>Median<br>(IQR)<br>(years)	Comorbidities (before COVID)	COVID-19<br>Date and duration<br><br>Severity	*Diagnosis	Blood markers	Management/<br>Treatment	Any other complications	How long follow up<br><br>Outcome	vaccination status	QA
			lower abdomen and suprapubic area<br>Tachycardiac (110–130 beats/ min) with a grade II–III systolic murmur over the apical area<br>Distended abdomen<br>Less bowel sounds<br>Hepatosplenomegaly<br>Scrotal, lower limb edema				presentation		ceftriaxone and vancomycin)<br><br>IVIG<br><br>Methylpred<br><br>Low-dose aspirin<br><br>Enoxaparin for venous thromboembolism prophylaxis	normal coronaries			
								Hgb 9.8 g/dL					
								Platelets 95.6×10^9/L					
								BUN 33mg/dL					
								Albumin 23 g/L					
								LDH 232 IU/L					
								Ferritin 748 ng/mL					
								BNP 13369 pg/mL					

Study	Study Type<br><br>Country	N (total)<br>Gender (%M or F)	MIS clinical features/ symptoms	Age Mean ±SE/ Median (IQR) (years)	Comorbidities (before COVID)	COVID-19 Date and duration<br><br>Severity	*Diagnosis	Blood markers	Management/ Treatment	Any other complications	How long follow up<br><br>Outcome	vaccination status	QA
								IL-6 26.8 0 pg/mL					
								PCT 2.87 ng/mL					
								ALP 614 IU/L					
								GGT 114 IU/L					
								Troponon T 0.083 ng/mL					
								CRP 140.7 mg/L					
								D-Dimer 2319 ng/mL					
								C3 complement 0.42 g/L					
								C4 <0.02 g/L					
Parpas et al. [39]	Case report, America	N=1<br>100% M	After 3 weeks of discharge from COVID treatment, he returned with generalized weakness, anorexia, weight loss, nausea, dyspnea, lower extremity	67 year old male	Past medical history of cirrhosis and HTN	COVID-19 pneumonia 68 days prior to admission requiring oxygen via high flow nasal cannula<br><br>Treated with doxycycline, ceftriaxone, vitamin C,	After 3 weeks post-discharge from COVID-19	WBC<br><br>Admission 1 5.9K/cmm	Broad-spectrum antibiotic did not correct leukocytosis (all cutlures -ve)<br><br>High D-dimer and clinical	See clinical characteristics and management/treatment	Rapid improvement in symtpoms and significat reduction in inflammatory markers after 15 days of steroid initation	Vaccination status not given	5 Murad et al.

Study	Study Type<br><br>Country	N (total)<br>Gender<br>(%M or F)	MIS<br>clinical features/<br>symptoms	Age<br>Mean ±SE/<br>Median<br>(IQR)<br>(years)	Comorbidities (before COVID)	COVID-19<br>Date and duration<br><br>Severity	*Diagnosis	Blood markers	Management/<br>Treatment	Any other complications	How long follow up<br><br>Outcome	vaccination status	QA
			swelling, and cognitive difficulties<br><br>Admitted with tachycardia, leukocytosis, AKI and severe hyponatremia 151/98mmHG, 115bpm, RR 20breaths/min, afebrile O2 sat 96% on room air Clear lungs, edma in all extremities and generalized muscle weakness<br><br>CXR showed bibasilar infiltrates and CT showed atelectasis<br><br>Urinalysis showed protein 100mg/dL, moderate blood, 10WBC/hpf, 5 RBC/hpf and muddy brown casTS<br><br>COVID PCR -ve but +ve antibodies<br><br>ECG showed grade 1 diastolic dysfunction, elevated pulmonary pressures, and normal LVEF			hydroxychloroquine, zinc, therapeutic enooxaprin, thiamin, dexamethasone, and convalescent plasma (for COVID treatment, not MIS-A			suspicion for pulmon ary emoblus led to administration of therapeutic unfractioned heparin and thrombotic evaluation<br><br>No pulmonary embolism and negative for deep vein thrombosis<br><br>Renal function deteriorated so HD was initiated<br><br>Renal biopsy showed moderate to marked acute tubular necrosis (ATN), no significant fibrosis, inflammation, immune complexes or viral inclusions		Normalized appetite, gained 10 pounds and was able to walk 15 feet BNP and inflammatory markers decreased		

Study	Study Type<br><br>Country	N (total)<br>Gender<br>(%M or F)	MIS<br>clinical features/<br>symptoms	Age<br>Mean ±SE/<br>Median<br>(IQR)<br>(years)	Comorbidities (before COVID)	COVID-19<br>Date and duration<br><br>Severity	*Diagnosis	Blood markers	Management/<br>Treatment	Any other complications	How long follow up<br><br>Outcome	vaccination status	QA
									Since patient was never hypotensive and AKI occurred before hospitalization, ATN was attributed to a cytokine surge<br><br>Received 5 HD treatments which led to renal improvement<br><br>Elevated inflammatory markers continued so dexamethasone 6mg/d was started for concern of MIS-A. After 4d, he showed improvement and was discharged on a prednisone taper				
								Neutrophils<br><br>Admission 1<br>63.6%					

Study	Study Type<br><br>Country	N (total)<br>Gender<br>(%M or F)	MIS<br>clinical features/<br>symptoms	Age<br>Mean ±SE/<br>Median<br>(IQR)<br>(years)	Comorbidities (before COVID)	COVID-19<br>Date and duration<br><br>Severity	*Diagnosis	Blood markers	Management/<br>Treatment	Any other complications	How long follow up<br><br>Outcome	vaccination status	QA
								Lymphocytes<br>:<br><br>Admission 1<br>19.5%					
								Hgb<br><br>Admission 1<br>16.6mg/dL					
								Platelets<br><br>Admission 1<br><br>325K/cmm					
								Sodium<br><br>Admission 1<br>136 mEq/L					
								Creatinine<br>Admission 1<br>1.1mg/dL					
								Albumin<br><br>Admission 1<br>3g/dL					
								D-dimer<br><br>Admission 1<br>3626ng/mL					

Study	Study Type<br><br>Country	N (total)<br>Gender<br>(%M or F)	MIS<br>clinical features/<br>symptoms	Age<br>Mean ±SE/<br>Median<br>(IQR)<br>(years)	Comorbidities (before COVID)	COVID-19<br>Date and duration<br><br>Severity	*Diagnosis	Blood markers	Management/<br>Treatment	Any other complications	How long follow up<br><br>Outcome	vaccination status	QA
								LDH<br>Admission 1<br><br>531U/L					
								BNP<br><br>Admission 1<br>19pg/mL					
								CRP<br><br>Admission 1<br>244.9mg/L					
								ESR<br><br>Admission 1<br>45mm/hr					
								Ferritin<br><br>Admission 1<br>1094.6ng/mL					
								Spot urine protein to creatinine ratio:<br>Admission 2<br>1.76					
								PCT:Admission 1<br>0.62ng/mL					

Study	Study Type<br><br>Country	N (total)<br>Gender<br>(%M or F)	MIS<br>clinical features/<br>symptoms	Age<br>Mean ±SE/<br>Median<br>(IQR)<br>(years)	Comorbidities (before COVID)	COVID-19<br>Date and duration<br><br>Severity	*Diagnosis	Blood markers	Management/<br>Treatment	Any other complications	How long follow up<br><br>Outcome	vaccination status	QA
Amato et al. [17]	Case Report<br><br>US	N=1<br>100	Fever<br>Conjunctivitis	25	Autism	PCR -ve<br>Antibodies positive during inpatient stay	During infection<br><br>30d	WBC 11.5*10^3 /uL	Colchicine<br>Steroids<br>Antibiotics<br><br>IVIG	Retrocardiac airspace opacity suspicious for pneumonia	Discharged after 17 days inpatient stay	NR	4<br>Murad et al.
								PCT <0.06 ng/mL					
								Lactate 0.8 mmol/L					
								ESR 123 mm/h					
								Hb 10.7 g/dL					
								CRP 22.1 mg/dL					
Tabatabai et al. [42]	Case Report<br><br>UAE	N=1<br>100% M	Shortness of breath<br>Chest discomfort<br>Orthopnea<br>Tachycardia	28	NR	Admitted for it 5 weeks earlier<br><br>Discharged in 4 days	During infection<br><br>1 week	Troponin 4302 ng/L	Oxygen<br>Enoxaparin<br>Furosemide<br>Prednisolone<br>Bisoprolol<br>Ramipril<br>Spironolactone	Acute myocarditis<br>Heart failure	Discharged 1 week later on meds	Not unvaccinated	4<br>Murad et al.
								TSH 3.6 uIU/mL					
								IL-6 17 pg/mL					
								SGPT 433 U/L					

Study	Study Type<br><br>Country	N (total)<br>Gender<br>(%M or F)	MIS<br>clinical features/<br>symptoms	Age<br>Mean ±SE/<br>Median<br>(IQR)<br>(years)	Comorbidities (before COVID)	COVID-19<br>Date and duration<br><br>Severity	*Diagnosis	Blood markers	Management/<br>Treatment	Any other complications	How long follow up<br><br>Outcome	vaccination status	QA
								LDH 2,695 U/L					
								Creatinine 0.5 mg/dL					
								PCT 0.15 ng/mL					
								D-dimer 1.92 ug/mL					
								CRP 100.3 mg/L					
								CK 532 ng/mL					
								Ferritin 559.4 ng/mL					

***How long after infectin or recovery**

AKI: Acute Kidney Injury; **ALP:** Alkaline Phosphatase; **ALC:** Absolute Lymphocyte Count; **ALT:** Alanine Aminotransferase; **AMS:** Altered Mental Status; **ANA:** Anti Nuclear Antibody; **ANCA:** Anti Neutrophil Cytoplasmic Antibody; **AST:** Aspartate Aminotransferase; **BNP:** B-Type Natriuretic Peptide; **CK:** Creatinine Kinase; **CRP:** C-Reactive Protein; **DBili:** Direct Bilirubin; **DM:** Diabetes Mellitus; **ESR:** Erythrocyte Sedimentation Rate; **GGT:** Gamma Glutamyl Transferase; **HD:** Hemodialysis; **HTN:** Hypertension; **IL:** Interleukin; **INR:** International Normalized Ratio; **IVIG:** Intravenous Immunoglobulin; **LDH:** Lactate Dehydrogenase; **LMWH:** Low Molecular Weight Heparin; **LVEF:** Left Ventricular Ejection Fraction; **MPO:** Myeloperoxidase; **PCT:** Procalcitonin; **PT:** Prothrombin Time; **PTT:** Partial Thromboplastin Time; **RBC**: Red Blood Cell; **RF:** Rheumatoid Factor; **SD:** Standard Deviation; **SGOT:** Serum glutamic oxaloacetic transaminase; **SGPT:** Serum glutamate pyruvate transaminase; **TBili:** Total Bilirubin; **TLC:** Total Lymphocte Count; **TSH:** Thyroid Stimulating Hormone; **US:** Ultra Sound; **WBC:** White Blood Cell Count
